# Orosomucoid 1 promotes colorectal cancer progression and liver metastasis by affecting PI3K/AKT pathway and inducing macrophage M2 polarization

**DOI:** 10.1038/s41598-023-40404-1

**Published:** 2023-08-28

**Authors:** Lei Yue, Xiaozhang Xu, Shipeng Dai, Fan Xu, Wenhu Zhao, Jian Gu, Xinzheng Dai, Xiaofeng Qian

**Affiliations:** 1 Zhejiang Cancer Hospital, Hangzhou Institute of Medicine (HIM), Chinese Academy of Sciences, Hangzhou, 310022 Zhejiang China; 2 Hepatobiliary Center, The First Affiliated Hospital of Nanjing Medical University, Nanjing, 210029 Jiangsu Province China; 3 Key Laboratory of Liver Transplantation, Chinese Academy of Medical Sciences, Nanjing, 210029 Jiangsu Province China; 4 NHC Key Laboratory of Living Donor Liver Transplantation, Nanjing Medical University, Nanjing, 210029 Jiangsu Province China

**Keywords:** Cancer, Gastrointestinal cancer, Metastasis

## Abstract

Approximately 25–30% of those affected by colorectal cancer (CRC), the most prevalent gastrointestinal malignancy, develop metastases. The survival rate of patients with liver metastasis of CRC (CRLM) remains low owing to its unpredictability and a lack of biomarkers that can be applied to distinguish groups at higher risk for CRLM among patients with CRC. Therefore, our study aimed to find biomarkers that can predict the risk of CRLM. Screening of the Gene Expression Omnibus database, supported by an analysis of clinically obtained tissue and serum data using qPCR and ELISA, in an attempt to identify relevant biomarkers, enabled us to determine that orosomucoid 1 (ORM1) was differentially expressed in liver metastases and primary tumors of patients with CRC. Functionally, overexpression of ORM1 promoted the epithelial-mesenchymal transition and the proliferative, migratory, and invasive activities of MC38 cells and activated the PI3K/AKT signaling pathway. Moreover, MC38 cells overexpressing ORM1 enhanced the tumor immune microenvironment by promoting macrophage M2 polarization and elevating interleukin-10 (IL-10) expression. In vivo experiments further confirmed in vitro results, indicating that liver metastases elevated by ORM1 were partially attenuated by the depletion of macrophages or IL-10. Considered together, ORM1 promotes CRC progression and liver metastasis by regulating tumor cell growth and inducing macrophage M2 polarization, which mediates tumor immune tolerance, and thus acts as a potential predictive marker and therapeutic target in CRLM.

## Introduction

As one of the most common forms of gastrointestinal cancer, colorectal cancer (CRC) is related to a high risk of recurrence and metastasis, and ranks second in terms of cancer-related death globally^1,2^. Due to its anatomical location, the liver becomes the most common site of metastasis for colorectal cancer^3^. Compared with patients without liver metastasis (LM), those with colorectal liver metastasis (CRLM) are characterized by significantly lower 5-year survival rates, which are often less than 20%^4,5^. The primary treatment for CRLM is surgical resection; however, more than 75% of patients experience postoperative recurrence^6^. Thus, the identification of biomarkers that is capable of indicating the possibility of developing CRLM, which is associated with poorer CRC prognoses, may be considered essential. Such biomarkers would help ensure the timely initiation of prophylactic anti-tumor therapy.

Orosomucoid (ORM), an acute-phase protein, also known as alpha-1-acid glycoprotein (AGP), has a molecular weight of approximately 44 kDa. It is mainly synthesized and released into the blood by hepatocytes^7^, and is categorized into two subtypes, ORM1 and ORM2^8^, which increase in response to stressful conditions, such as inflammation, infection, injury, and burns^9–11^. The expression of ORM1 varies depending on the histological origin of different malignancies. For example, elevated ORM1 levels seen in patients with breast cancer leads to tumorigenesis and epirubicin resistance^12,13^. In addition, in hepatocellular carcinoma, ORM1 is activated and acts as an antitumor protein which decreases tumors^14^. Furthermore, in colorectal cancer, ORM1 is believed to be a hub gene associated with CRLM^15^. Nevertheless, the exact mechanism by which ORM1 participates in CRLM remains to be elucidated.

In this study, public datasets and clinical results enabled us to determine that CRLM occurrence was associated with high ORM1 expression. Mechanistic data revealed that overexpression of ORM1 not only enhanced the epithelial-mesenchymal transition (EMT) as well as the proliferative and migratory activities of tumor cells but also accelerated macrophage M2 polarization and IL-10 expression, which induced the formation of an immunosuppressive microenvironment that further promoted tumor growth. The findings of our study may be considered important because we identified a molecule that is directly associated with CRLM as well as with poor prognoses for CRC. In addition, high serum ORM1 expression was observed in patients with CRLM. In brief, ORM1 shows potential as both a therapeutic target and predictive marker for CRLM.

## Materials and methods

### Microarray dataset

Based on the GEO database (https://www.ncbi.nlm.nih.gov/geo/)^16^, gene expression data were obtained for normal colon tissues, liver metastases, and primary tumors of CRC. Three mRNA datasets (GSE14297, GSE49355, and GSE81558) containing 34 normal colon tissues, 56 liver metastases, and 61 primary CRC tumors were selected and analyzed.

### The determination of differentially expressed genes (DEGs)

GEO2R (https://www.ncbi.nlm.nih.gov/geo/geo2r/), an online data-processing tool in GEO based on R, was used to analyze DEGs between liver metastases and primary tumors of CRC. Inclusion criteria for DEGs was |log fold change (FC)|> 1 and *p* < 0.05.

### Analysis of DEGs enriched by gene ontology (GO) and Kyoto encyclopedia of genes and genomes (KEGG)

The cell components (CC), molecular functions (MF), and biological processes (BP) associated with DEGs were identified using GO functional analysis. The enrichment analysis of KEGG pathway^17–19^ was conducted to identify biological pathways related to DEGs. The cutoff criteria used for these analyses were gene counts > 5 and p.adjust < 0.05.

### Establishment of a protein–protein interaction (PPI) network and determination of hub genes

By using the Search Tool for Retrieval of Interacting Genes (STRING) (http://string-db.org)^20^, the PPI network of DEG-encoded proteins was established and visualized via Cytoscape software (version 3.9.0)^21^. Identification of hub genes was performed by using the cytoHubba plug-in. The top 20 most dysregulated genes, calculated using the degree method, were selected as hub genes and subjected to KEGG pathway enrichment analysis.

### Kaplan–Meier (KM) survival analyses of hub genes

To understand how hub genes affect the prognosis in CRC, high- or low-expression groups were separated by using the median of hub gene expression as the boundary. Survival analysis was performed with a KM curve via the gene expression profiling interactive analysis (GEPIA) server tool (http://gepia.cancer-pku.cn/)^22^. *p* < 0.05 was set as the significance threshold.

### Clinical specimens

A total of 20 CRC tissues (10 from patients with CRC and 10 from patients with CRLM) and 10 liver metastases of CRC, along with their matched adjacent normal tissues, were collected from the First Affiliated Hospital of Nanjing Medical University, Nanjing, China. Human blood samples were obtained from CRC patients (n = 10), CRLM patients (n = 10), healthy people (n = 10), patients with benign liver diseases [focal nodular hyperplasia (FNH, n = 10) and hepatic hemangioma (n = 10)] or malignant tumors [intrahepatic cholangiocarcinoma (ICC, n = 10) and hepatocellular carcinoma (HCC, n = 10)], and patients with CRLM before (n = 10) and after (n = 10) partial hepatectomy. The plasma was stored at − 80 °C after being separated from the whole blood. Selected patients had no other malignant tumors. Informed consent was obtained from all participants and/or their legal guardians while approval was obtained from the Ethics Committee of the First Affiliated Hospital of Nanjing Medical University (Approval NO: 2021-SRFA-424). All methods were performed in accordance with the relevant guidelines.

### Cell culture

Human colon cancer cell lines HCT116 (non-metastatic colon cancer cells, ZQ0125), SW480 (moderately metastatic colon cancer cells, ZQ0063), Lovo (highly metastatic colon cancer cells, ZQ0058), and the mouse colon adenocarcinoma cell line MC38 (ZQ0933) were purchased from Shanghai Zhong Qiao Xin Zhou Biotechnology Co. Ltd (Shanghai, China). HCT116, SW480, and MC38 cells were cultured in DMEM (KGM12800-500, Keygen Biotech, Nanjing, China) with 10% fetal bovine serum (FBS, 164210-50, Procell, Wuhan, China). Lovo cells were cultured in Ham’s F-12K medium (G4560-500ML, Servicebio, Wuhan, China) supplemented with 10% FBS. All cells were maintained in a 5% CO_2_-containing incubator (Thermo Fisher Scientific, Waltham, MA, USA) at 37 °C.

### Cell transfection

MC38 cells were transfected with an ORM1 mimic or negative control (NC) mimic (HZC05014, Genepharma, Shanghai, China). Transfection was performed according to the manufacturer's instructions. RNA and proteins were extracted from cells after 48 h to verify the effect of transfection.

### Rearing of experimental mice

Male C57BL/6 mice (6–8 weeks old, GemPharmatech, Nanjing, China) were selected for these experiments, and housed under the following conditions: constant humidity; a temperature of 22 °C; a 12 h/12 h alternating light/dark cycle that simulated day and night; individually ventilated rearing cages, and unlimited dietary water.

### CRLM mouse model establishment

Animal studies were conducted according to the Guidelines for the Care and Use of Laboratory Animals and approved by the Ethics Committee of Nanjing Medical University (Approval NO: 2022-SRFA-317). The study is reported in accordance with ARRIVE guidelines (PLoS Bio 8(6), e1000412, 2010). After anesthesia, the transverse incision was made in the upper left abdomen to open abdominal cavity and the spleen was separated and exposed. Next, a 1 ml syringe was used to inject 100 µl MC38 cells at a concentration of approximately 1 × 10^7^ cells/ml into the splenic capsule at the lower pole of the spleen. When the splenic capsule at the injection site was white and swollen, the needle was pulled out, and the bleeding was stopped by pressure for 2 min. The abdomen was then closed layer by layer. Liver tissues were harvested after 3 weeks, and tumor numbers were recorded. The sample size for each experimental group was three.

Subsequently, clodronate-filled liposomes (CLD, 40337ES08, Yeasen Biotechnology, Shanghai, China) or the IL-10 inhibitor, AS101 (sc-203825, Santa Cruz Biotechnology, Dallas, TX, USA), were administered before the construction of CRLM mouse models. One day before modeling, 200 µl of CLD was injected into each mouse via intraperitoneal injection at 3-day intervals, whereas 10 µg of AS101 was intraperitoneally injected every day until the end of modeling.

### Bone marrow-derived macrophages (BMDMs) extraction and culture

After cervical dislocation, the femurs and tibia of the mice were separated and attached tissues were removed. Next, bone marrow was flushed into the centrifuge tube with DMEM containing 10% FBS and centrifuged at 1250 rpm for 5 min. Subsequently, the supernatant was removed, treated with 3 ml medium and red blood cell lysate, mixed, and centrifuged again under the same conditions. The final step was to resuspend the cells in DMEM for culture after the supernatant was removed.

### Co-cultured system

To induce macrophage polarization, BMDMs isolated from mice were co-cultured with the supernatant of the MC38 cells, which had been transfected with lentivirus overexpressing ORM1 or the negative control for 3 days. In addition, the functional markers of M1 or M2 macrophages were assayed.

### qPCR

A total RNA extraction kit (RN001, Yishan Biotech, Shanghai, China) was applied to extract RNA from liver metastases, primary tumors of CRC, or colon cancer cells. The concentration of RNA was detected by Nanodrop spectrophotometer (Thermo Fisher Scientific). The first complementary DNA (cDNA) strand was synthesized using the GoScript Reverse Transcription System (Promega, Madison, Wisconsin, WI, USA). Next, qPCR analyses (10 µl) were conducted according to the ratio of pure water: SYBR Green (Q141-02, Vazyme, Nanjing, China): pre-primer: post-primer: cDNA = 3.6:5.0:0.2:0.2:1.0. The relative RNA levels were estimated using a 2^−∆∆Ct^ method. Primer sequences that were used (Sangon Biotech, Shanghai, China) are listed in Table 1. GAPDH was used as an internal reference.

**Table 1:** The gene-specific primer sequences.

Primer name	Primer sequences-forward	Primer sequences-reverse
ORM1	5’-CTGACAAGCCAGAGACGACCAA-3’	5’-TGCTTCTCCAGTGGCTCACACT-3’
CD86	5’-ATGGGCTCGTATGATTGT-3ʹ	5’-CTTCTTAGGTTTCGGGTG-3ʹ
NOS2	5’-CACAGCAATATAGGCTCATCCA-3’	5’-GGATTTCAGCCTCATGGTAAAC-3’
ARG1	5’-AGGCGCTGTCATCGATTTCT-3’	5’-TGGAGTCCAGCAGACTCAAT-3’
MRC1	5’-GTTCACCTGGAGTGATGGTTCTC-3’	5’-AGGACATGCCAGGGTCACCTTT-3ʹ
GAPDH	5’- GTCTCCTCTGACTTCAACAGCG-3’	5’-ACCACCCTGTTGCTGTAGCCAA-3’

### ELISA assays

ORM1, TGF-β, IL-1β, IL-10, and TNF-a levels in tissues or cell supernatants were detected by commercially available ELISA kits (MultiSciences, Hangzhou, China).

### Western blot analysis

Cells that had been treated were collected and total proteins were extracted with RIPA buffer (G2002-100ML, Servicebio). Bicinchoninic Acid (BCA) method was applied to detect protein concentrations. Equivalent proteins were separated via 10% SDS-PAGE (PG111, Epizyme, Shanghai, China) and then transferred to PVDF membranes (IPVH00010, Millipore, Burlington, MA, USA). Next, incubation with primary antibodies overnight was performed at 4 °C after the membranes were blocked with a 5% skim milk powder solution at room temperature for 1 h. Following rinsing in Tris Buffered Saline-Tween (TBST, G0004-500ML, Servicebio), the membranes were incubated for 2 h at room temperature with HRP-labeled secondary antibodies (1:2000, PR30011, Proteintech, Wuhan, China). Primary antibodies against Vimentin (Vim, 1:2000, 10366-1-AP, Proteintech), N-cadherin (N-Ca, 1:2000, 22018-1-AP, Proteintech), E-cadherin (E-Ca, 1:2000, 20874-1-AP, Proteintech), ORM1 (1:1000, 16439-1-AP, Proteintech), phosphorylated PI3K (p-PI3K, 1:2000, ab278545, Abcam, Waltham, MA, USA), PI3K (1:1000, ab151549, Abcam), phosphorylated AKT (p-AKT, 1:1000, ab38449, Abcam), AKT (1:500, ab8805, Abcam), and GAPDH (1:5000, 10494-1-AP, Proteintech) were used. All antibodies were diluted in the proper proportions using an antibody diluent (WB050D, NCM Biotech, Suzhou, China).

### Immunofluorescence assay

Paraffin sections of liver metastases from mice were dewaxed, antigen repaired, and serum blocked. The sections were incubated overnight at 4 °C with primary antibodies, followed by incubation at room temperature for 1 h with secondary antibodies. After washing with PBS (C10010500BT, Gibco, Waltham, MA, USA) thrice, the sections were incubated at room temperature for 10 min with bovine serum albumin (GC305010-100g, Servicebio) in the dark, and then washed thrice with TBST. Afterward, sections were reverse-stained with 4',6-diamidino-2-phenylindole (G1012-10ML, Servicebio) and sealed. Finally, a fluorescent microscope (Zeiss, Oberkochen, Germany) was applied to observe the images.

### Cell counting kit‐8 (CCK‐8) assay

Transfected MC38 cells were plated at approximately 3000 cells/ well into the 96-well plate. Five replicates were established for each group. Next, 10 µl CCK-8 reagent (PM508, Dojindo Laboratories, Japan) was added to every well and incubated at 37 °C for 2 h at different time points (24, 48, 72, or 96 h), after which a microplate reader (Thermo Fisher Scientific) was applied to detect the absorbance at 450 nm.

### Colony formation assay

First, each well of the 6-well plate was inoculated with 1000 MC38 cells and then incubated in at 37 °C 5% CO_2_ for 14 d until colonies were visible. Thereafter, the colonies were washed with PBS, fixed with 4% paraformaldehyde (G1101, Servicebio) for half an hour, washed again with PBS, and stained with crystal violet (C0121-100 ml, Beyotime Biotechnology, Shanghai, China) for 30 min. After staining, each well was photographed, and ImageJ software (National Institutes of Health, Bethesda, MD, USA) was used to calculated colony numbers.

### Wound healing assay

Approximately 8 × 10^5^ MC38 cells were plated into each well of the 6-well plate. When cell density reached about 90%, 10 μl sterile pipette was applied to create scratches on cell monolayer. Open wound areas were photographed using a microscope at 0, 24, or 48 h. Finally, the percentage reduction of the open wound area relative to the initial scratch (0 h) was analyzed at different time points by using ImageJ software.

### Transwell assay

Transwell chambers (3422; Corning Inc., Corning, NY, USA) placed into a 24-well plate were used to perform a Transwell assay. First, 2 × 10^4^ MC38 cells were plated into the upper chamber and cultured with 200 µl serum-free DMEM. Next, 800 µl DMEM+20% FBS was added to the lower chamber. The Transwell chambers were then washed with PBS after incubating at 37 °C for 24 h, fixed with 4% paraformaldehyde for 30 min, washed again with PBS, and stained with crystal violet for half an hour. Each Transwell chamber was photographed with a microscope, and the amount of stained cells in the image was measured by using Image J software.

### Statistical analysis

All statistical analyses were conducted using GraphPad Prism 9.0 (GraphPad Software, San Diego, CA, USA). All data are expressed as mean ± standard deviation (SD) and analyzed using Student’s *t*-test for comparison between two groups and one-way analysis of variance (ANOVA) for univariate comparison. Pearson’s correlation analysis was applied to measure the correlation between two different variables. Statistical significance was set at ns *p* ≥ 0.05, *p < 0.05, **p < 0.01, ***p < 0.001, and ****p < 0.0001.

## Results

### DEGs between LM and primary tumors (PT) of CRC

**Figure 1:**
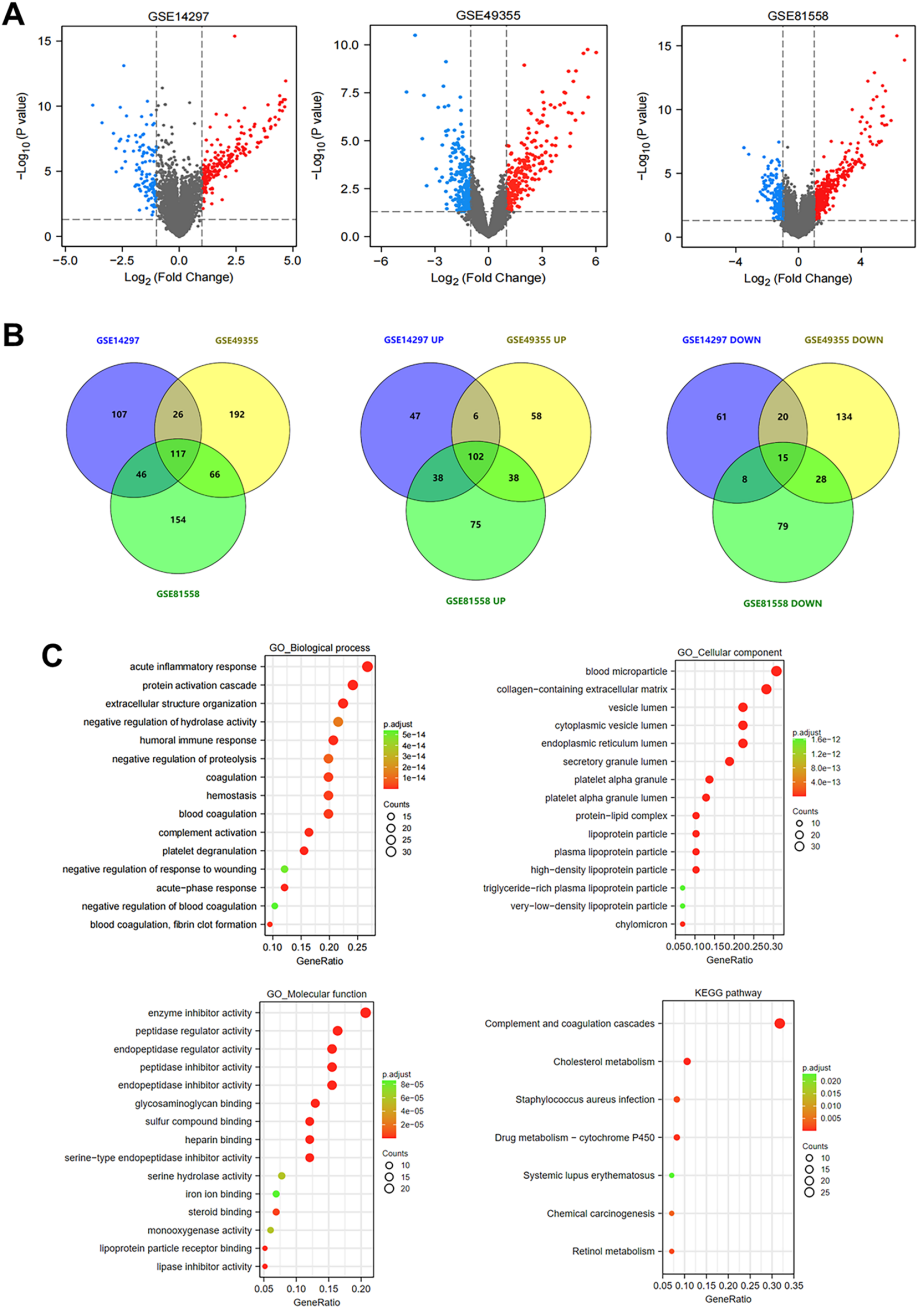
Identification of DEGs between liver metastasis and primary CRC samples in datasets GSE14297, GSE49355, and GSE81558 (|log_2_FC|> 1 and *P*-value < 0.05). (**A**) Volcano plots for DEGs. The red color represents the upregulated genes, while the blue color represents the downregulated genes, and the black indicates no significant difference between primary CRC tumors and liver metastases. (**B**) Venn plots for consistent total DEGs, upregulated DEGs, and downregulated DEGs. (**C**) GO (Biological process, Cellular component, and Molecular function) and KEGG pathway enrichment analysis of consistent DEGs.

To explore the mRNA expression levels associated with CRLM, we analyzed three datasets from the GEO database to determine dysregulated mRNA expression between LM and PT of CRC. A total of 56 LM and 61 PT expression data from GSE14297, GSE49355, and GSE81558, respectively, were included in this study (Fig. S1A). Based on |log_2_FC|> 1 and *p* < 0.05, 117 common DEGs (102 upregulated and 15 downregulated genes) were determined in these three datasets (Fig. 1A,B). Specific DEGs are shown in Fig. S1B. GO functional analysis revealed that these DEGs were obviously related to acute inflammatory response, blood microparticles, and enzyme inhibitor activity in terms of BP, CC, and MF, respectively. KEGG enrichment of DEGs mainly involved complement and coagulation cascades and cholesterol metabolism (Fig. 1C).

### Construction of PPI network and hub gene determination

**Figure 2:**
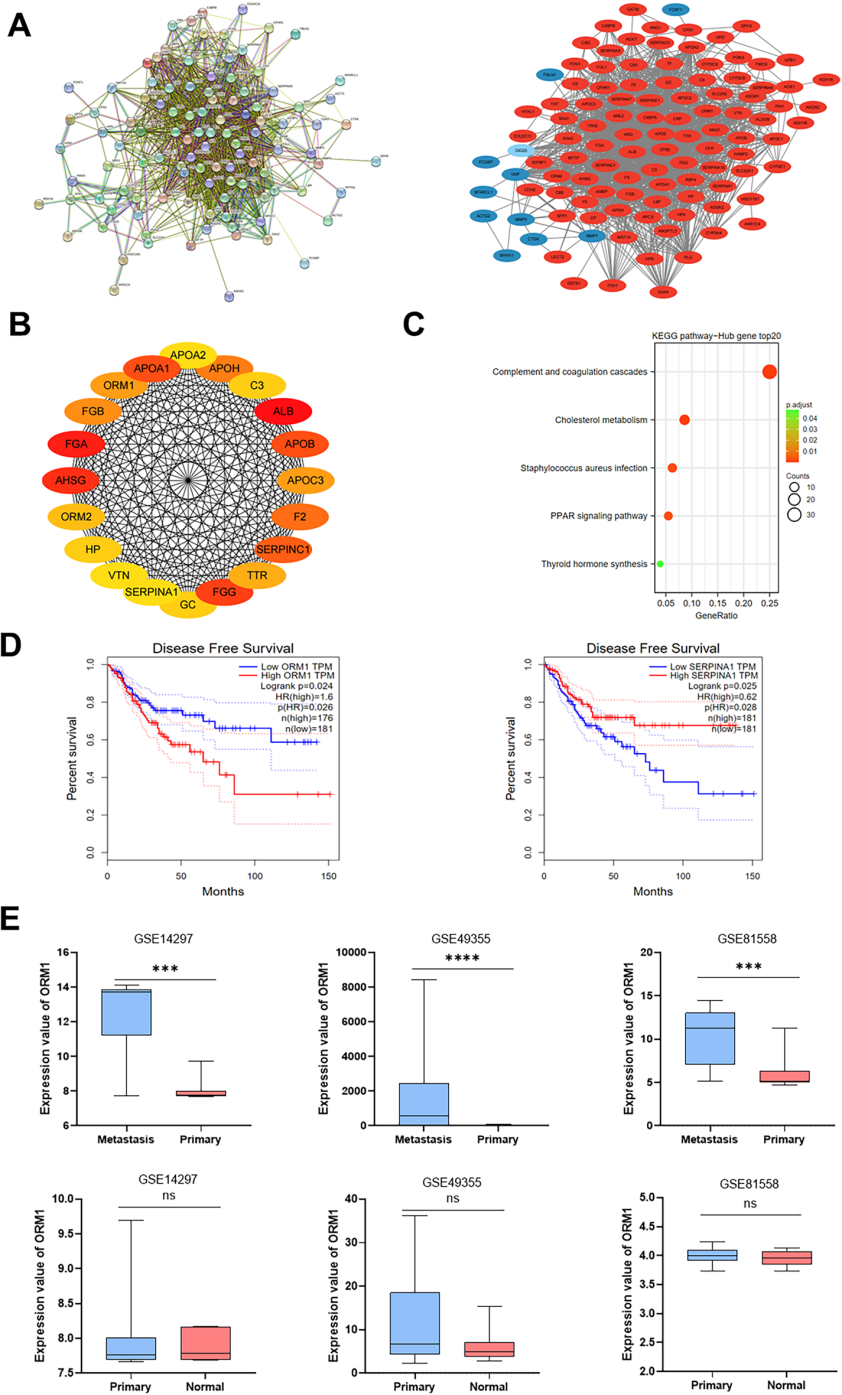
(**A**) PPI network of DEGs was established by STRING (http://string-db.org) and visualized via Cytoscape software (version 3.9.0). The red nodes indicated upregulated genes and the blue nodes indicated downregulated genes. (**B**) Top 20 hub genes with a higher degree of connectivity. (**C**) KEGG pathway enrichment analysis of the top 20 hub genes. (**D**) The prognostic value (*p* < 0.05) of the top 20 hub genes in CRC patients in the disease-free survival (DFS) curve (GEPIA). (**E**) The expression of ORM1 between liver metastases and primary tumors, primary tumors, and normal tissues of CRC in datasets GSE14297, GSE49355, and GSE81558 respectively. Data are expressed as mean ± SD. ns *p* ≥ 0.05, ***p < 0.001, ****p < 0.0001 (unpaired t test or ANOVA).

A PPI network including 117 nodes and 1347 edges was established by using STRING and Cytoscape software (Fig. 2A). Top 20 hub genes were determined by using the cytoHubba plugin and sorted according to their degree of connectivity (Fig. 2B, S2A). KEGG pathway analysis indicated that PPAR signaling pathways, cholesterol metabolism, and complement and coagulation cascades were primarily enriched in the top 20 hub genes (Fig. 2C).

Next, we investigated the correlation between the top 20 hub genes and CRC prognosis by using GEPIA. The KM survival analysis indicated that ORM1 overexpression was related to poor disease-free survival (DFS) (*p* = 0.024), whereas high SERPINA expression predicted a longer DFS (*p* = 0.025), contrasting with its expression trend in LM and PT of CRC (Fig. 2D, S2B). Thus, under these circumstances, ORM1 expression acted as a poor prognostic factor.

Furthermore, we compared ORM1 expression among LM, PT of CRC, and normal colorectal tissues by analyzing datasets GSE14297, GSE49355, and GSE81558. Compared with that in CRC tissues, the levels of ORM1 were higher in CRLM, whereas there was no clear difference between the ORM1 levels of CRC tissues and normal colorectal tissues (Fig. 2E). This result suggested that ORM1 may contribute to CRC progression and liver metastasis, rather than CRC occurrence.

### ORM1 expression is upregulated in LM of CRC and shows the potential predictive value for CRLM

**Figure 3:**
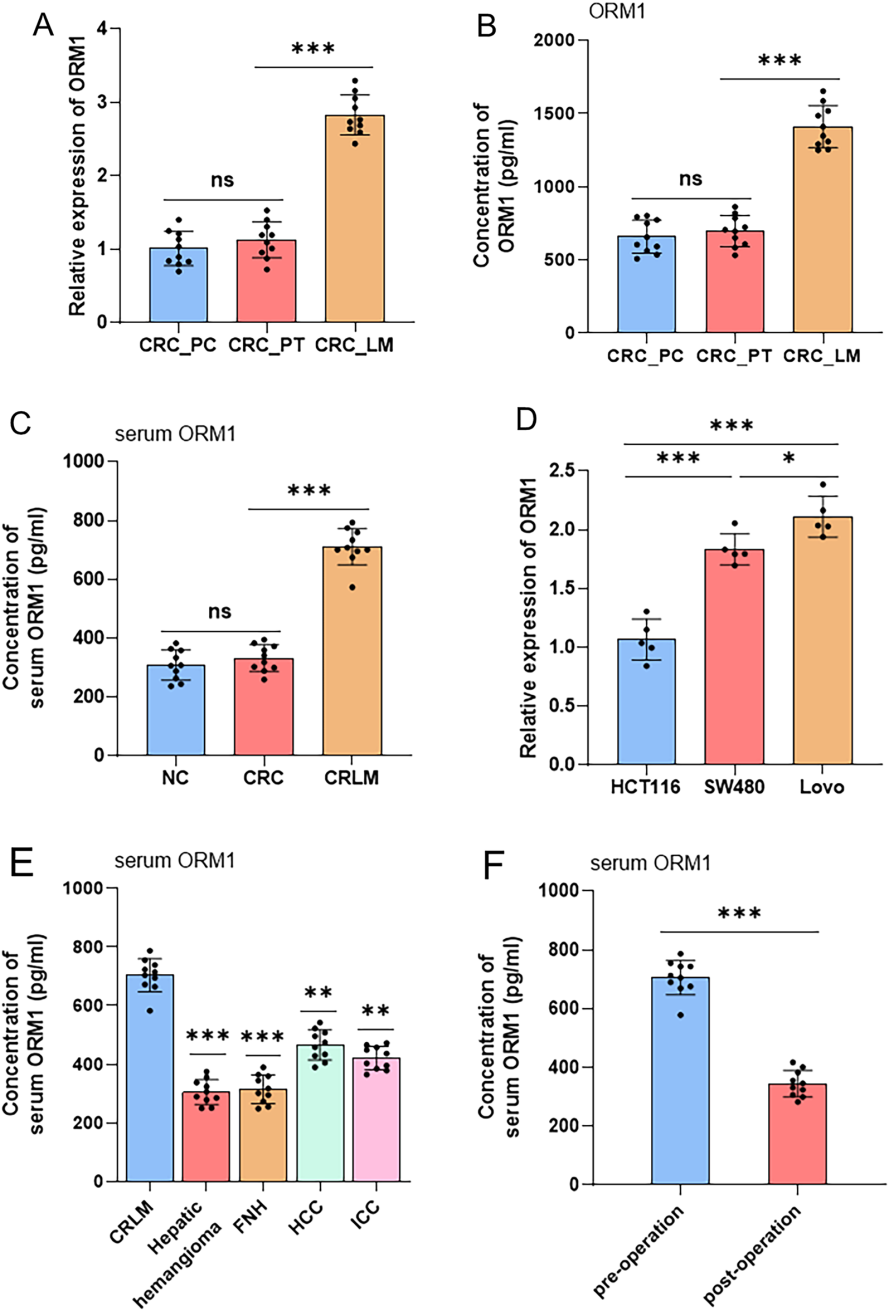
(**A**) The expression level of ORM1 mRNA in the liver metastases, primary tumors of CRC patients, and the adjacent normal tissues detected by qPCR. (**B**) The protein concentrations of ORM1 in the liver metastases, primary tumors, and their para-cancerous tissues of CRC measured by ELISA. (**C**) Serum concentrations of ORM1 in CRC patients with or without liver metastasis and healthy individuals. (**D**) The levels of ORM1 mRNA expressed in human colon cancer cell lines HCT116, SW480, and Lovo. (**E**) Serum concentrations of ORM1 in patients with benign liver diseases (hepatic hemangioma and FNH) or malignant tumors (CRLM, HCC, and ICC). (**F**) Serum concentrations of ORM1 in CRLM patients before and after surgery. Data are expressed as mean ± SD. ns *p* ≥ 0.05, *p < 0.05, **p < 0.01, ***p < 0.001 (unpaired t test or ANOVA).

To further validate the difference between the expression levels of ORM1 in LM and PT of CRC, we collected 10 pairs of PT tissues and LM and their para-cancer (PC) tissues of CRC from the First Affiliated Hospital of Nanjing Medical University. ORM1 expression was evaluated using qPCR and ELISA. The levels of ORM1 in LM (CRC_LM) were upregulated compared with that in PT (CRC_PT), whereas there were no significant differences between that in CRC-PT and its PC tissues (CRC-PC) (Fig. 3A,B). Moreover, we measured serum ORM1 levels in CRC patients with or without LM, and healthy individuals, using ELISA. The results also indicated that high levels of serum ORM1 were found in CRLM patients, whereas no clear differences were found between CRC patients and normal subjects (Fig. 3C). In addition, we examined ORM1 levels in CRC cell with different metastatic potentials. Compared with that in non-metastatic colon cancer cell HCT116, moderately or highly metastatic colon cancer cells, SW480 or Lovo, highly expressed ORM1 (Fig. 3D). Considered together, ORM1 levels were upregulated both in serum and LM of CRLM patients and CRC cell lines with metastatic potential.

We further investigated the predictive role of ORM1 in CRLM by detecting ORM1 expression levels in 10 blood samples from patients with benign liver diseases (hepatic hemangioma and FNH) or malignant tumors (HCC and ICC) using ELISA. Serum ORM1 levels were significantly higher in CRLM patients than those in patients with benign liver diseases (hepatic hemangioma or FNH), HCC, or ICC (Fig. 3E). Furthermore, blood samples were obtained on third day after surgical resection of their liver metastases, to assess changes in ORM1 before and after surgery. These results showed that serum ORM1 levels significantly decreased after surgery (Fig. 3F), indicating that serum ORM1 may be of potential diagnostic and predictive value in relation to CRLM.

### ORM1 promotes CRLM and mediates macrophage M2 polarization

**Figure 4:**
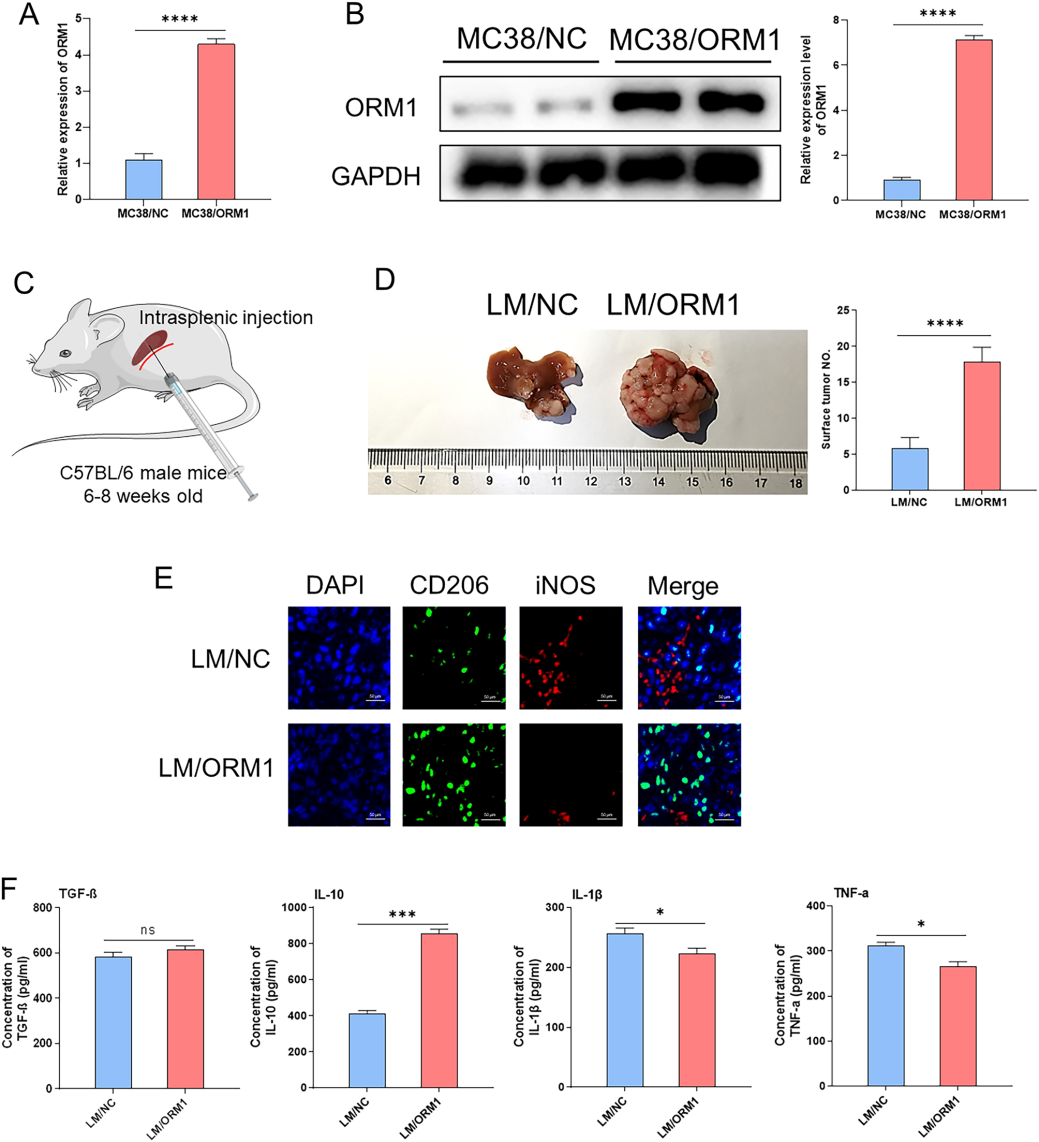
(**A**,**B**) qPCR (**A**) and western blot (**B**) analysis of ORM1 overexpression in MC38 cells. (**C**) A mouse model of CRLM was constructed by intrasplenic injection of ORM1-overexpressing MC38 or control cells. (**D**,**E**) Metastatic nodules of the liver (**D**) and representative images of immunofluorescence staining for CD206 and iNOS from metastatic lesions by (**E**) are shown. (**F**) The expression levels of TGF-β, IL-10, IL-1β, and TNF-a in metastatic nodules of the liver were measured by ELISA. Data are expressed as mean ± SD. ns *p* ≥ 0.05, *p < 0.05, ***p < 0.001, ****p < 0.0001 (unpaired t test or ANOVA).

We transfected lentivirus overexpressing ORM1 or empty vector into MC38 cells (MC38/ORM1 or MC38/NC) and measured transfection efficiency via qPCR and western blotting. ORM1 was stably overexpressed in MC38/ORM1 cells (Fig. 4A,B). Subsequently, mouse CRLM models were established via intrasplenic injection of MC38 cells into C57BL/6 mice (Fig. 4C). After four weeks, livers were harvested and inspected for the number of LM on their surfaces. The detectable tumor numbers in the LM of mice treated with MC38/ORM1 cells (LM/ORM1) were higher than those in mice treated with MC38/NC cells (LM/NC); (Fig. 4D), indicating that upregulation of ORM1 was effective in promoting CRLM.

Macrophages, the most abundant non-parenchymal cells in the liver, are closely associated with tumor immunity. Their polarization into M1 or M2 subtype affects the proliferation and metastasis of tumor cells^23^. Therefore, we used immunofluorescence (IF) staining to monitor macrophage polarization in liver metastases. Our results revealed that compared with LM/NC, the M2 macrophage marker, CD206, was elevated, and the M1 marker, iNOS, was decreased in LM/ORM1 (Fig. 4E), thereby demonstrating that ORM1 may mediate macrophage M2 polarization.

Furthermore, the levels of some macrophage-associated cytokines, IL-1β and TNF-a, mainly produced by M1, as well as IL-10 and TGF-β, usually secreted by M2, were analyzed using ELISA. The result was similar to that of IF; the level of IL-10 was elevated, whereas TNF-a and IL-1β were slightly reduced in LM/ORM1 compared with LM/NC. However, no significant difference was detected in TGF-β concentration between the two groups (Fig. 4F). Collectively, these results indicated that ORM1 overexpression may promote CRLM and induce macrophage M2 polarization.

### ORM1 induces EMT, promotes the proliferative, migratory, and invasive activities of colon cancer cells, and affects the PI3K/AKT pathway

**Figure 5:**
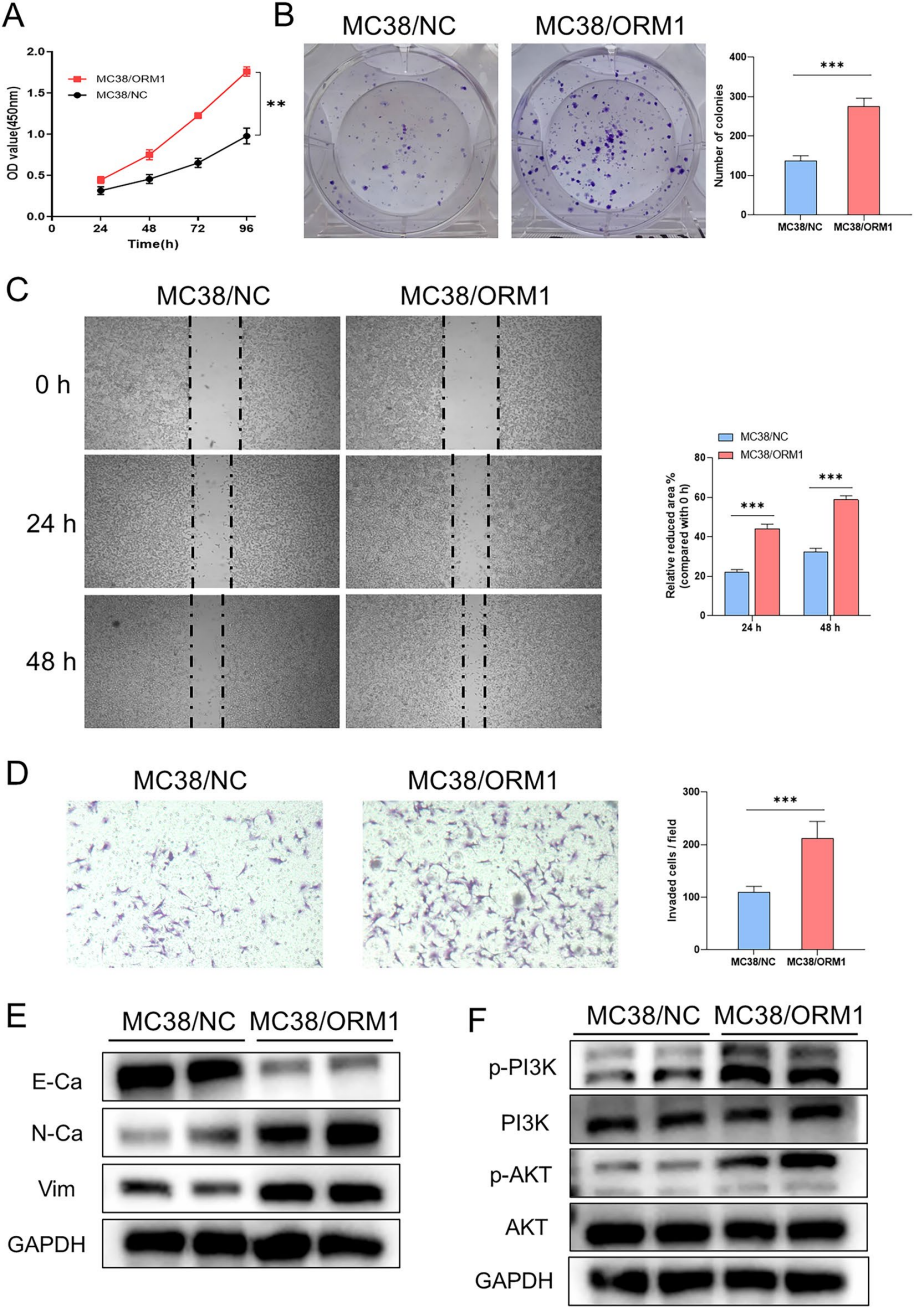
(**A**) CCK8 assays were used to detect cell proliferation at 24, 48, 72, and 96 h in ORM1-overexpressing MC38 or control cells. (**B**) Representative images and statistical results of colony formation assays. (**C**) Analysis of MC38 cells overexpressing ORM1 or control migration by wound-healing assays at 0, 24, and 48 h. Statistical results are shown in the right panel. (**D**) The migration of ORM1-overexpressing MC38 or control cells was assessed using Transwell migration assays and the statistical results. (**E**) The protein levels of EMT-related markers E-cadherin (E-Ca), N-cadherin (N-Ca), and Vimentin (Vim) in ORM1-overexpressing MC38 cells were analyzed by western blot. (**F**) Western blot assay was performed to detect the level of p-PI3K, PI3K, p-AKT, and AKT. Data are expressed as mean ± SD. **p < 0.01, ***p < 0.001 (unpaired t test or ANOVA).

The malignant progression of tumors is a complex process. EMT and the proliferative, migratory, and invasive activities of cancer cells, are important events in tumorigenesis and development. To investigate how ORM1 promotes CRC progression and liver metastasis, MC38 cells transfected with lentivirus were used for in vitro experiments. CCK-8 and colony formation assays were applied to evaluate cell proliferation. The proliferation rate and the number of colonies of MC38/ORM1 cells were much higher than those of MC38/NC cells (Fig. 5A,B). Wound healing and Transwell assays were performed to evaluate the migratory and invasive activities. After 24 and 48 h of culture, the migration area (compared with that at 0 h) of MC38/ORM1 cells was larger than that of MC38/NC cells (Fig. 5C). The invasion ability showed similar tendency; MC38/ORM1 cells showed a greater invasive capacity than MC38/NC cells (Fig. 5D). Hence, it was concluded that high levels of ORM1 promoted the proliferative, migratory, and invasive activities of colon cancer cells.

EMT is a critical biological process, which enables cancer cells to invade and metastasize^24,25^. Therefore, we analyzed the proteins, E-Ca, Vim, and N-Ca, that are associated with EMT in MC38 cells. Western blotting confirmed that mesenchymal phenotype markers, N-Ca and Vim, were upregulated, whereas epithelial phenotype marker E-Ca was downregulated in MC38/ORM1 cells, in contrast to MC38/NC cells (Fig. 5E, S3A). This implies that ORM1 overexpression may promote the transformation of MC38 cells from the epithelial phenotype to the more invasive and metastatic mesenchymal phenotype.

Furthermore, we investigated how ORM1 promotes the malignant phenotype of CRC cells. The PI3K/AKT pathway, a key intracellular signaling pathway regulating the malignant potential of cancer cells, plays a vital role in many malignancies, including ovarian^26^, gastric^27^, and breast cancers^28^. Therefore, we used western blot to measure protein expression in the PI3K/AKT signaling pathway. Our results indicated that p-PI3K and p-AKT levels were significantly increased in MC38 cells overexpressing ORM1, while total-PI3K and AKT expression remained unaltered (Fig. 5F, S3B). In summation, these results demonstrated that ORM1 induces malignant phenotypes in colon cancer cells by affecting PI3K/AKT signaling pathway.

### ORM1 facilitates CRLM partially by inducing macrophage M2 polarization

In our previous study, elevated ORM1 levels were found in the peripheral blood and liver metastases of CRLM patients. Furthermore, an increased proportion of M2 macrophages was observed in the liver metastasis of mice injected with MC38/ORM1. Therefore, we investigated whether ORM1 facilitates CRLM by inducing macrophage M2 polarization.

**Figure 6:**
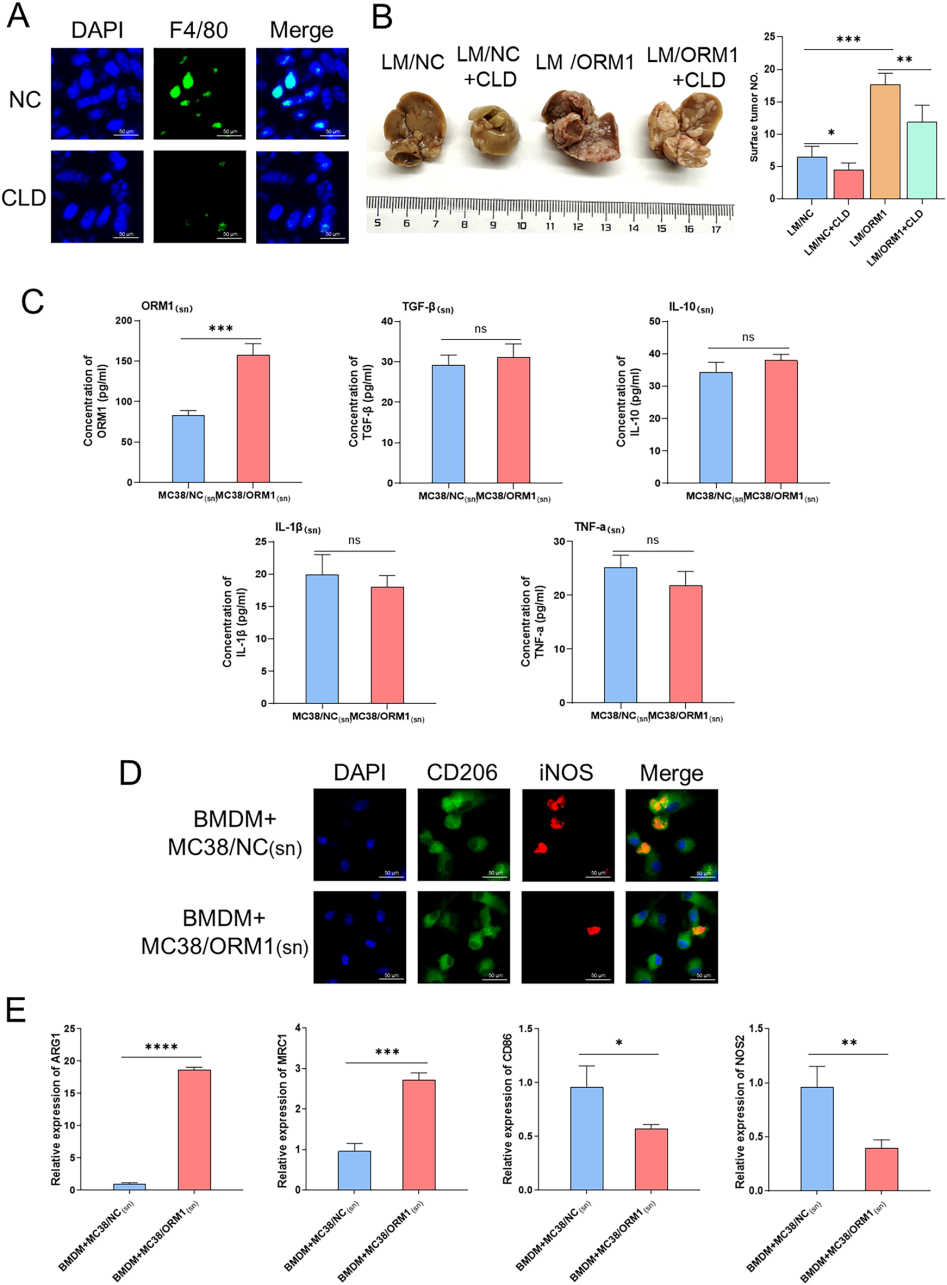
(**A**) Immunofluorescence staining for F4/80 in liver metastases from control and CLD-treated mice. (**B**) Representative images of liver metastases from the MC38/NC or MC38/ORM1-injected mice treated with CLD or not. Statistical results of the detectable tumor numbers of liver metastasis are shown in the right panel. (**C**) The levels of ORM1, TGF-β, IL-10, IL-1β, and TNF-a in the supernatant of MC38/ORM1 or MC38/NC cells measured by ELISA. (**D**) Immunofluorescence staining for CD206 and iNOS from BMDMs co-cultured with MC38/ORM1_(sn)_ or MC38/NC_(sn)_. (**E**) Representative histograms of the relative mRNA levels of ARG1, MRC1, CD86, and NOS2 for BMDMs treated with MC38/ORM1_(sn)_ or MC38/NC_(sn)_. Data are expressed as mean ± SD. ns *p* ≥ 0.05, *p < 0.05, **p < 0.01, ***p < 0.001, ****p < 0.0001 (unpaired t test or ANOVA).

Clodronate-filled liposomes (CLD) were used in in vivo experiments to deplete macrophages^29^. We intraperitoneally inoculated mouse CRLM models with CLD. Immunofluorescence staining indicated that the expression of F4/80, a macrophage marker, was notably reduced in liver metastases of CLD treated mice (Fig. 6A). Compared with that in mice inoculated with MC38/ORM1 cells alone (LM/ORM1), the detectable number of liver metastases was decreased to a certain extent in mice treated with MC38/ORM1 cells and CLD (LM/ORM1+CLD), whereas compared with the liver metastases of the LM/NC group, those of the LM/NC+CLD group were slightly reduced (Fig. 6B). These results indicated that ORM1 may partially facilitate CRLM by inducing macrophage M2 polarization.

Moreover, in vitro studies were conducted to further confirm the promotion of macrophage M2 polarization by ORM1. We co-cultured BMDMs isolated from mice with the supernatants of MC38/ORM1 or MC38/NC cells [MC38/ORM1_(sn)_ or MC38/NC_(sn)_]. Before co-culture, we measured the levels of ORM1 and some cytokines in MC38/ORM1_(sn)_ and MC38/NC_(sn)_ via ELISA. The ORM1 levels in MC38/ORM1_(sn)_ were higher than that in MC38/NC_(sn)_, whereas no clear difference was found in cytokine levels between the two groups, including TNF-a, IL-10, TGF-β, and IL-1β (Fig. 6C). Next, the BMDMs were co-cultured with the supernatant. After 3 days, we determined the polarization of BMDMs using IF and qPCR. The IF indicated that, compared with that in the group co-cultured with MC38/NC_(sn)_, the expression of CD206 was increased whereas that of iNOS was downregulated in the group co-cultured with MC38/ORM1_(sn)_ (Fig. 6D). Furthermore, qPCR showed that the expression of M2 markers, ARG1 and MRC1, were increased, while the levels of M1 markers, CD86 and NOS2, were decreased in BMDMs co-cultured with MC38/ORM1_(sn)_ (Fig. 6E). In conclusion, the findings above illustrated that ORM1 may be a mediator of macrophage polarization into M2 phenotype.

### ORM1 promotes the secretion of pro-tumor-associated cytokines by macrophages

**Figure 7:**
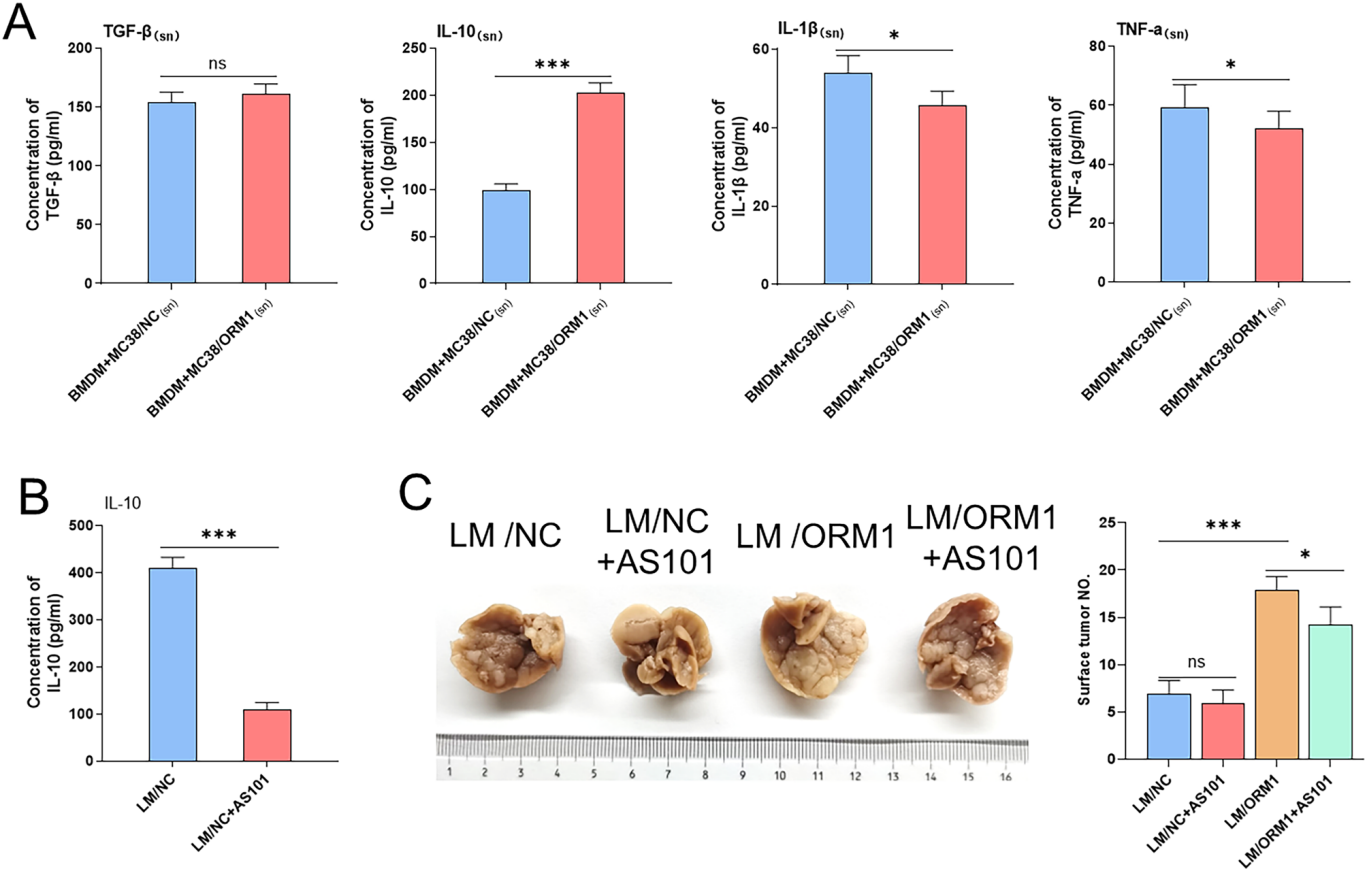
(**A**) The levels of TGF-β, IL-10, IL-1β, and TNF-a in the supernatant of BMDMs after co-cultured with MC38/ORM1_(sn)_ or MC38/NC_(sn)_ measured by ELISA. (**B**) Representative histograms of the concentration of IL-10 in liver metastases from CRLM model mice treated with AS101 or not. (**C**) The detectable tumor numbers in liver metastasis from the MC38/NC or MC38/ORM1-injected mice treated with AS101 or not. Data are expressed as mean ± SD. ns *p* ≥ 0.05, *p < 0.05, ***p < 0.001 (unpaired t test or ANOVA).

After co-culture, we examined the cytokines secreted mainly by M1 or M2 macrophages in the supernatant. Much higher levels of IL-10 were found in MC38/ORM1_(sn)_ after co-culturing with BMDMs, whereas the concentration of TGF-β was slightly increased without a statistical difference between the two groups. The TNF-a and IL-1β levels were mildly reduced (Fig. 7A). Hence, we hypothesized that ORM1 may promote CRLM in part by regulating macrophage M2 polarization which contributes to the secretion of associated pro-tumor cytokines.

IL-10, one of the main anti-inflammatory cytokines produced by M2 macrophages, plays a significant part in regulating immune response and tumor progression^30,31^. Therefore, we investigated the role played by the IL-10 inhibitor, AS101, an immunomodulator that effectively inhibits IL-10 mRNA transcription, in the association between ORM1 on CRLM. According to the research conducted by Kalechman et al., the optimal AS101 dose for inhibiting IL-10 transcription in mice was 10 µg^32^. Therefore, we used this dose for subsequent experiments. The inhibitory effect of AS101 on IL-10 was confirmed by an ELISA of liver metastases (Fig. 7B). Although detectable tumor numbers in the liver metastases of the LM/ORM1 group were somewhat reduced when treated with AS 101, no significant difference was found in the detectable tumor numbers of the LM/NC group, whether treated with AS101 or not (Fig. 7C). Overall, these results indicated that IL-10 shows a positive effect on the promotion of CRLM by ORM1.

## Discussion

CRC is the third most common form of cancer^33–35^. Progression from CRC to CRLM worsens prognoses several folds and contributes to many more deaths from CRC. Currently, diagnosis of CRLM is based on colonoscopy, abdominal imaging (such as hepatic B ultrasound, enhanced abdominal CT, or MRI), serum carcinoembryonic antigen (CEA), and needle biopsy^36^. However, these indexes are often associated with disadvantages stemming from hysteresis. Hence, understanding the molecular mechanisms underlying CRLM progression, identifying key oncogenes that regulate migration, invasion, and liver metastasis of cancer cells, and screening for molecular markers that predict the risk of developing CRLM, may be considered essential for monitoring the development of CRLM and designing new therapeutic strategies against it.

In the current study, data from GEO database illustrated that ORM1 expression was increased in liver metastasis compared to that in the primary tumor of CRC, although there was no significant difference between primary tumors and adjacent tissues of CRC. These results were further validated by examining clinical tissue samples. In addition, the finding indicating that the serum ORM1 levels of patients with CRLM were higher, as opposed to the absence of a clear difference between patients with CRC and normal subjects, suggested that ORM1 shows potential as a biomarker of CRLM. The established CRLM models indicated that ORM1 may promote liver metastasis by mediating macrophage M2 polarization and related cytokine secretion. Functional assays revealed that upregulation of ORM1 not only enhanced proliferative, migratory, and invasive activities but also promoted the EMT of CRC cells and affected the PI3K/AKT pathway. Co-culture of BMDMs with the supernatant of MC38 cells overexpressing ORM1 also promoted macrophage M2 polarization. Finally, the stimulatory effect of ORM1-mediated macrophage M2 polarization on CRLM was confirmed via in vivo experiments using CLD, or AS101. Hence, ORM1 may be considered as both a predictor of CRLM and a potential target for immunotherapy^37^.

ORM1 is commonly recognized as an acute-phase protein that is released into the blood by hepatocytes under stressful conditions, such as chronic inflammation, injury, or infection^9,10^. By contrast, recent studies have demonstrated that *ORM1* may show a vital role as an oncogene in several cancers. Zhou et al. indicated that serum ORM1 might serve as a predictor of lower therapeutic response to chemotherapy and poor prognosis in advanced extranodal NK/T-cell lymphoma^38^. In addition, Choi et al.^12^ noted that ORM1 levels were elevated in patients with breast cancer, particularly in those resistant to epirubicin^13^. Similar results have been reported in ovarian cancer^39^ and lung adenocarcinoma^40^. These findings all support the application of ORM1 as a biomarker of therapeutic efficacy and prognosis in tumors, which is in accordance with our findings. Some studies have also demonstrated the diagnostic value of ORM1 in other cancers. For example, Zhan et al. reported that urinary ORM1 combined with a-fetoprotein (AFP) effectively improved the diagnosis of HBV-associated hepatocellular carcinoma (HCC)^41^. A recent study showed that ORM1 increases vascular invasion and reduces sensitivity to sorafenib in HCC^42^. Similarly, serum ORM1 and TGF-β levels may be useful as reliable clinical biomarkers in the early diagnosis of non-small cell lung cancer^43^. In addition to acting on cancer cells, ORM1 acts on the immune system. Matsusaka et al. indicated that AGP activates intracellular STAT1 by binding to CD14, a co-receptor for toll-like receptor 4 (TLR4) on macrophages, thereby inducing programmed death ligand 1 (PD-L1) expression and producing IL-6, which in turn promotes tumor progression^37^. Another study by Nakamura noted that ORM1 induces the polarization of quiescent monocytes into M2b macrophages, which may be beneficial in controlling opportunistic infections^44^. These findings demonstrate that ORM1 stimulates a shift in immune cells towards a pro-tumor phenotype, which, to some extent, corroborates its role as an oncogene. In our study, upregulation of ORM1 promoted macrophage M2 polarization and secreted M2-associated cytokines. These cytokines may create an immune microenvironment in the liver that is conducive to tumor cell colonization and growth, thereby creating favorable conditions for CRLM. ORM1 also affects intracellular signaling pathways. For example, the PI3K/AKT pathway is a key signaling pathway that regulates the malignant potential of cancer cells by activating or inhibiting downstream molecules^45,46^. Previous studies have found that a large proportion of factors that influence CRC progression and metastasis act via the PI3K/AKT pathway^47–49^. Our study also indicates that ORM1 overexpression facilitates the phosphorylation of PI3K and AKT. Furthermore, W922, a novel PI3K/AKT pathway inhibitor, showed efficient antitumor effects on CRC cells^50^. However, its role in CRLM remains unclear, indicating the need for future studies.

Our study illustrated that ORM1 shows potential as a marker of CRLM and may be utilized to clinically predict the risk of CRLM occurrence as well as to diagnose CRLM. On the one hand, serum ORM1 may be useful for assessing the risk of CRLM occurrence in CRC patients who undergo regular reviews following radical resection. On the other hand, when smaller hepatic occupancies are found and imaging examinations are unable to provide a valid judgment, the determination of serum ORM1 may provide additional information that may be useful in arriving at a definitive diagnosis. Considered together, ORM1 may act as a favored marker for monitoring and early diagnosis of CRLM. However, the current study conducted only a preliminary exploration of the potential mechanisms of ORM1 in CRLM and was affected by some limitations such as insufficient clinical samples and insufficient verification of experimental results. The exact mechanism underlying the role of ORM1 in PI3K/AKT signal-mediated CRLM remains unclear. Therefore, further studies may be warranted.

## Conclusions

Our study demonstrated that ORM1 levels were increased in the liver metastases of CRC, serum of patients with CRLM, and CRC cell lines with metastatic potential. Upregulation of ORM1 expression facilitated the malignant potential of CRC cells, affected the PI3K/AKT pathway, and mediated macrophage M2 polarization. And ORM1 may have the potential to be both a therapeutic target and predictive marker for CRLM.

### Supplementary Information

Supplementary Figures.

Supplementary Information.

## Data Availability

The datasets used and/or analyzed during the current study are available from the Gene Expression Omnibus (GEO, https://www.ncbi.nlm.nih.gov/geo/), accession number GSE14297, GSE49355 and GSE81558.

## References

[1] Keum N, Giovannucci E (2019). Global burden of colorectal cancer: Emerging trends, risk factors and prevention strategies. Nat. Rev. Gastroenterol. Hepatol..

[2] Siegel RL (2020). Colorectal cancer statistics, 2020. CA Cancer J. Clin..

[3] LeGolvan MP, Resnick M (2010). Pathobiology of colorectal cancer hepatic metastases with an emphasis on prognostic factors. J. Surg. Oncol..

[4] Jones RP (2016). Colorectal liver metastases: A critical review of state of the art. Liver Cancer.

[5] Bray F (2018). Global cancer statistics 2018: GLOBOCAN estimates of incidence and mortality worldwide for 36 cancers in 185 countries. CA Cancer J. Clin..

[6] Rees M, Tekkis PP, Welsh FK, O'Rourke T, John TG (2008). Evaluation of long-term survival after hepatic resection for metastatic colorectal cancer: A multifactorial model of 929 patients. Ann. Surg..

[7] Luo Z, Lei H, Sun Y, Liu X, Su DF (2015). Orosomucoid, an acute response protein with multiple modulating activities. J. Physiol. Biochem..

[8] Yuasa I (1993). Orosomucoid system: 17 additional orosomucoid variants and proposal for a new nomenclature. Vox Sang..

[9] Ligresti G, Aplin AC, Dunn BE, Morrishita A, Nicosia RF (2012). The acute phase reactant orosomucoid-1 is a bimodal regulator of angiogenesis with time- and context-dependent inhibitory and stimulatory properties. PLoS ONE.

[10] Astrup LB (2019). Staphylococcus aureus infected embolic stroke upregulates Orm1 and Cxcl2 in a rat model of septic stroke pathology. Neurol. Res..

[11] Petersen HH, Nielsen JP, Heegaard PM (2004). Application of acute phase protein measurements in veterinary clinical chemistry. Vet. Res..

[12] Choi JW (2020). Serum levels and glycosylation changes of Alpha-1-acid glycoprotein according to severity of breast cancer in Korean women. J. Microbiol. Biotechnol..

[13] Qiong L, Yin J (2021). Orosomucoid 1 promotes epirubicin resistance in breast cancer by upregulating the expression of matrix metalloproteinases 2 and 9. Bioengineered.

[14] Zhu HZ (2020). Downregulation of orosomucoid 2 acts as a prognostic factor associated with cancer-promoting pathways in liver cancer. World J. Gastroenterol..

[15] Liu S (2021). Identification of hub genes related to liver metastasis of colorectal cancer by integrative analysis. Front. Oncol..

[16] Clough E, Barrett T (2016). The gene expression omnibus database. Methods Mol. Biol. (Clifton, N.J.).

[17] Kanehisa M, Goto S (2000). KEGG: Kyoto encyclopedia of genes and genomes. Nucleic Acids Res..

[18] Kanehisa M (2019). Toward understanding the origin and evolution of cellular organisms. Protein Sci. Publ. Protein Soc..

[19] Kanehisa M, Furumichi M, Sato Y, Kawashima M, Ishiguro-Watanabe M (2023). KEGG for taxonomy-based analysis of pathways and genomes. Nucleic Acids Res..

[20] Szklarczyk D (2019). STRING v11: Protein-protein association networks with increased coverage, supporting functional discovery in genome-wide experimental datasets. Nucleic Acids Res..

[21] Shannon P (2003). Cytoscape: A software environment for integrated models of biomolecular interaction networks. Genome Res..

[22] Tang Z (2017). GEPIA: A web server for cancer and normal gene expression profiling and interactive analyses. Nucleic Acids Res..

[23] Xia Y (2020). Engineering macrophages for cancer immunotherapy and drug delivery. Adv. Mater. (Deerfield Beach, Fla).

[24] Chen T, You Y, Jiang H, Wang ZZ (2017). Epithelial-mesenchymal transition (EMT): A biological process in the development, stem cell differentiation, and tumorigenesis. J. Cell. Physiol..

[25] Lu W, Kang Y (2019). Epithelial-mesenchymal plasticity in cancer progression and metastasis. Dev. Cell.

[26] Ediriweera MK, Tennekoon KH, Samarakoon SR (2019). Role of the PI3K/AKT/mTOR signaling pathway in ovarian cancer: Biological and therapeutic significance. Semin. Cancer Biol..

[27] Fattahi S, Amjadi-Moheb F, Tabaripour R, Ashrafi GH, Akhavan-Niaki H (2020). PI3K/AKT/mTOR signaling in gastric cancer: Epigenetics and beyond. Life Sci..

[28] Guerrero-Zotano A, Mayer IA, Arteaga CL (2016). PI3K/AKT/mTOR: Role in breast cancer progression, drug resistance, and treatment. Cancer Metastasis Rev..

[29] Bader JE (2018). Macrophage depletion using clodronate liposomes decreases tumorigenesis and alters gut microbiota in the AOM/DSS mouse model of colon cancer. Am. J. Physiol. Gastrointest. Liver Physiol..

[30] Liu Q (2020). Wnt5a-induced M2 polarization of tumor-associated macrophages via IL-10 promotes colorectal cancer progression. Cell Commun. Signal.

[31] Hambardzumyan D, Gutmann DH, Kettenmann H (2016). The role of microglia and macrophages in glioma maintenance and progression. Nat. Neurosci..

[32] Kalechman Y (2002). Anti-IL-10 therapeutic strategy using the immunomodulator AS101 in protecting mice from sepsis-induced death: Dependence on timing of immunomodulating intervention. J. Immunol. (Baltimore Md.: 1950).

[33] Siegel RL (2019). Global patterns and trends in colorectal cancer incidence in young adults. Gut.

[34] Lu B (2021). Colorectal cancer incidence and mortality: The current status, temporal trends and their attributable risk factors in 60 countries in 2000–2019. Chin. Med. J..

[35] Rawla P, Sunkara T, Barsouk A (2019). Epidemiology of colorectal cancer: Incidence, mortality, survival, and risk factors. Prz. Gastroenterol..

[36] Xu J (2019). Chinese guidelines for the diagnosis and comprehensive treatment of colorectal liver metastases (version 2018). J. Cancer Res. Clin. Oncol..

[37] Matsusaka K (2021). α(1)-acid glycoprotein enhances the immunosuppressive and protumor functions of tumor-associated macrophages. Can. Res..

[38] Zhou Z (2016). S100A9 and ORM1 serve as predictors of therapeutic response and prognostic factors in advanced extranodal NK/T cell lymphoma patients treated with pegaspargase/gemcitabine. Sci. Rep..

[39] Wu W (2016). Identification of proteomic and metabolic signatures associated with chemoresistance of human epithelial ovarian cancer. Int. J. Oncol..

[40] Xu YF, Xu Y, Li X, Yang XM (2018). Serum α-1 acid glycoprotein is a biomarker for the prediction of targeted therapy resistance in advanced EGFR-positive lung adenocarcinoma. Comb. Chem. High Throughput Screen..

[41] Zhan Z (2020). Urine α-fetoprotein and orosomucoid 1 as biomarkers of hepatitis B virus-associated hepatocellular carcinoma. Am. J. Physiol. Gastrointest. Liver Physiol..

[42] Gu J (2022). ORM 1 as a biomarker of increased vascular invasion and decreased sorafenib sensitivity in hepatocellular carcinoma. Bosn. J. Basic Med. Sci..

[43] Ye X (2020). Dramatically changed immune-related molecules as early diagnostic biomarkers of non-small cell lung cancer. FEBS J..

[44] Nakamura K, Ito I, Kobayashi M, Herndon DN, Suzuki F (2015). Orosomucoid 1 drives opportunistic infections through the polarization of monocytes to the M2b phenotype. Cytokine.

[45] Osaki M, Oshimura M, Ito H (2004). PI3K-Akt pathway: Its functions and alterations in human cancer. Apoptosis Int. J. Program. Cell Death.

[46] Fresno Vara JA (2004). PI3K/Akt signalling pathway and cancer. Cancer Treat. Rev..

[47] Ma Z, Lou S, Jiang Z (2020). PHLDA2 regulates EMT and autophagy in colorectal cancer via the PI3K/AKT signaling pathway. Aging.

[48] Jiang T (2021). CircIL4R activates the PI3K/AKT signaling pathway via the miR-761/TRIM29/PHLPP1 axis and promotes proliferation and metastasis in colorectal cancer. Mol. Cancer.

[49] Duan S (2018). IMPDH2 promotes colorectal cancer progression through activation of the PI3K/AKT/mTOR and PI3K/AKT/FOXO1 signaling pathways. J. Exp. Clin. Cancer Res. CR..

[50] Wang J (2021). Novel PI3K/Akt/mTOR signaling inhibitor, W922, prevents colorectal cancer growth via the regulation of autophagy. Int. J. Oncol..

