# Supplementary material for: Orosomucoid 1 promotes colorectal cancer progression and liver metastasis by affecting PI3K/AKT pathway and inducing macrophage M2 polarization
Source: Sci Rep. 2023 Aug 28;13:14092. doi: 10.1038/s41598-023-40404-1 (PMC10462626; doi:10.1038/s41598-023-40404-1)
Supplement: Supplementary file 1 — Supplementary Figures. [file 41598_2023_40404_MOESM1_ESM.pdf]

Fig S1

A

Dataset	Normal Colon Epithelium	Primary Tumor	Liver Metastases	Platform
GSE14297	7	18	18	GPL6370
GSE49355	18	20	19	GPL96
GSE81558	9	23	19	GPL15207

B

Regulation	DEGs
Upregulated (n=102)	CRP、FGA、ORM1、FGB、APOA1、ALB、HP、AMBP、GC、APOA2、TF、FGL1、CYP2E1、ORM2、VTN、F2、FGG、HPX、APOC3、AHSG、APOB、SERPINC1、HRG、KNG1、SERPINA3、APCS、LBP、CYP2C8、TTR、ITIH1、C4BPA、PLG、APOC1、ITIH2、AGXT、C6、HPD、SAA4、APOH、APOC2、C5、RBP4、C8A、FCN3、CP、F9、ASGR1、CPB2、CPS1、COLEC11、IGFBP1、CYP2C9、C8B、AOX1、C3、FMO3、SERPINA6、TAT、SERPINA5、ANGPTL3、ASGR2、APOE、HPR、SLC2A2、RDH16、C8G、ARG1、ALDOB、SERPIND1、SAA2、HSD11B1、HABP2、GATM、PAH、VNN1、SERPINA7、CYP3A4、AQP9、DPYS、F5、SPP1、PON3、CDH2、AKR1C4、MAT1A、UPB1、MBL2、SERPING1、SERPINA1、CDO1、CFH、LECT2、SERPINA10、CFHR1、SLC22A1、DEFB1、SAA1、C4BPB、HYAL1、MTTP、ADH1B、AADAC
Downregulated (n=15)	ANGPTL2、FBLN1、FOXF1、DES、MMP1、MYH11、GREM1、CTSK、VWF、SPARCL1、CXCL14、MMP3、ACTG2、FCGBP、SPINK4

Supplementary Figure 1. DEGs between LM and primary tumors (PT) of CRC

(A) Gene expression data extracted from three GEO datasets. **(B)** List of consistent DEGs, including 102 upregulated DEGs and 15 downregulated DEGs.

Fig S2

A

Rank	Name	Type	Score
1	ALB	up	74
2	FGA	up	63
3	AHSG	up	60
4	FGG	up	59
5	APOA1	up	58
5	APOB	up	58
7	SERPINC1	up	57
8	F2	up	56
9	APOH	up	55
10	FGB	up	54
11	APOC3	up	52
11	ORM1	up	52
13	TTR	up	51
14	ORM2	up	49
15	C3	up	48
15	HP	up	48
15	GC	up	48
18	VTN	up	47
18	APOA2	up	47
18	SERPINA1	up	47

B

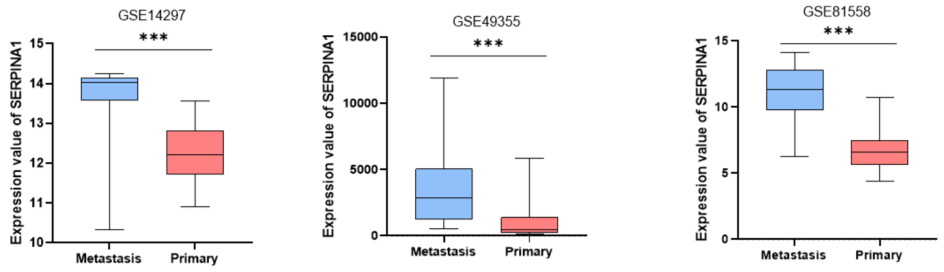

Supplementary Figure 2. Construction of PPI network and hub gene determination

(A) List of top 20 hub genes with a higher degree of connectivity in DEGs. **(B)** The expression of SERPINA1 between liver metastases and primary tumors of CRC in datasets GSE14297, GSE49355, and GSE81558 respectively. Data are expressed as mean ± SD. ***p <0.001 (unpaired t test or ANOVA).

Fig S3

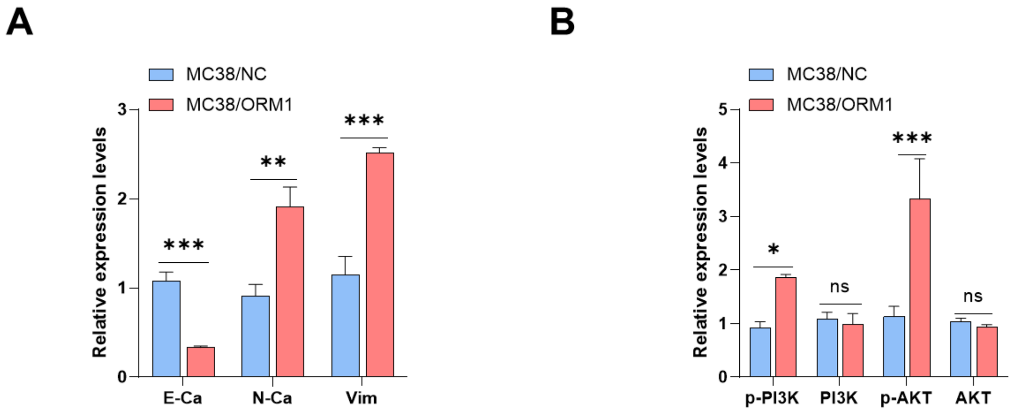

Supplementary Figure 3. ORM1 induces EMT, promotes the proliferative, migratory, and invasive activities of colon cancer cells, and affects the PI3K/AKT pathway

(A) The relative expression levels of EMT-related markers E-cadherin (E-Ca), N-cadherin (N-Ca), and Vimentin (Vim) in ORM1-overexpressing MC38 cells. (B) The relative expression levels of p-PI3K, PI3K, p-AKT, and AKT in ORM1-overexpressing MC38 cells. Data are expressed as mean ± SD. ns $p \geqslant 0.05$, *$p < 0.05$, **$p < 0.01$, ***$p < 0.001$ (unpaired t test or ANOVA).
