# Supplementary figures and images for: Orosomucoid 1 promotes colorectal cancer progression and liver metastasis by affecting PI3K/AKT pathway and inducing macrophage M2 polarization
Source: Sci Rep. 2023 Aug 28;13:14092. doi: 10.1038/s41598-023-40404-1 (PMC10462626; doi:10.1038/s41598-023-40404-1)

Fig 4B

GAPDH

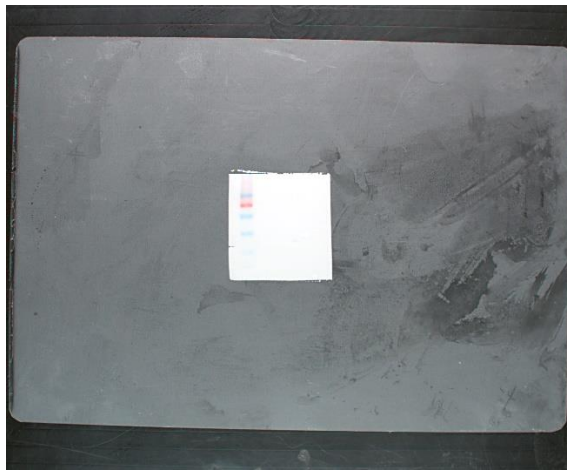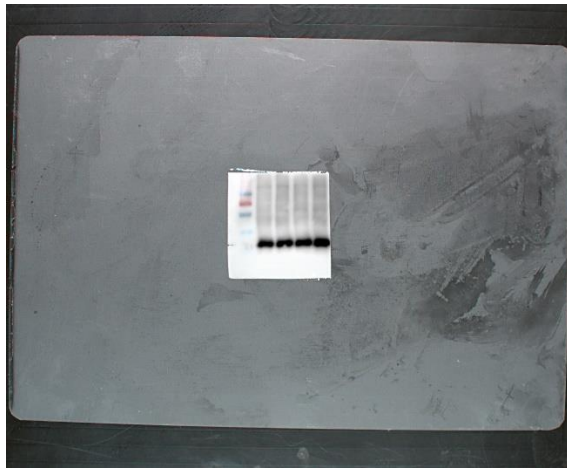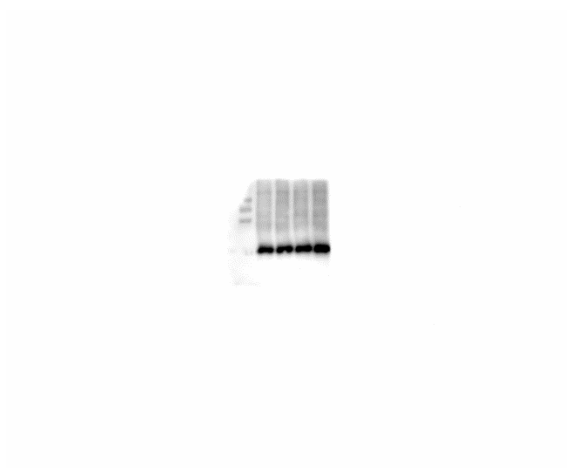

ORM1

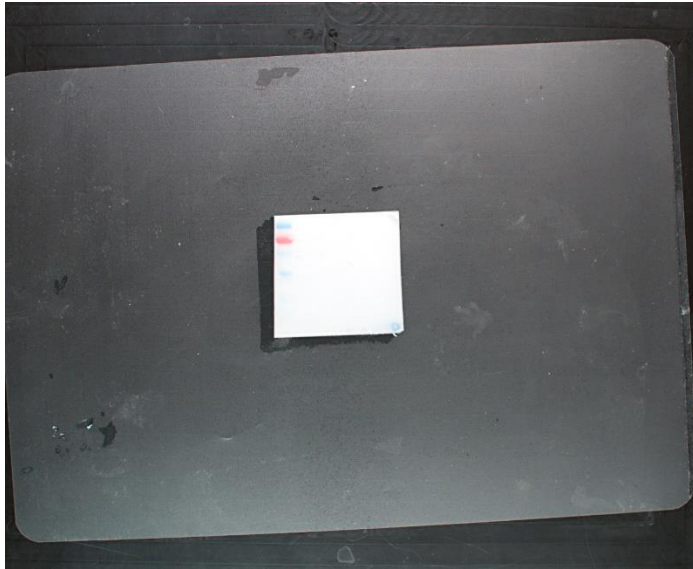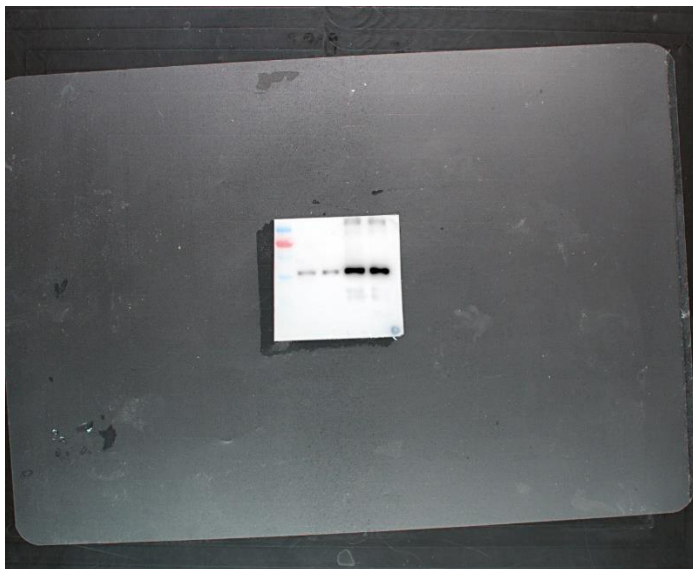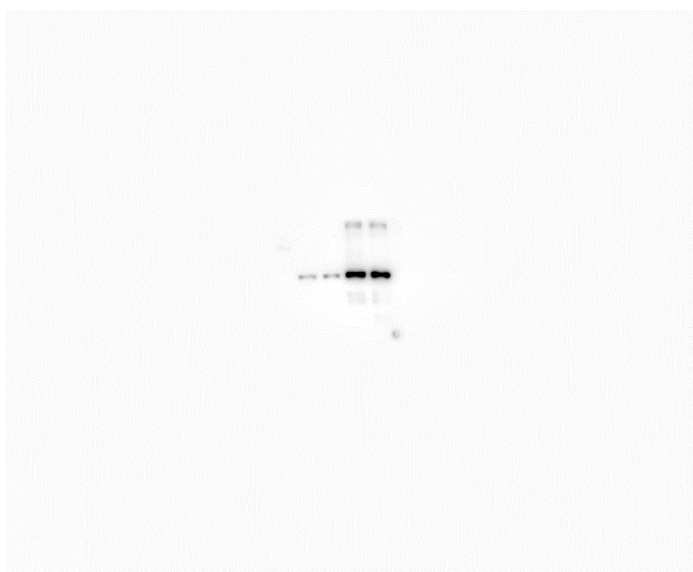

Fig 5E

E-Ca

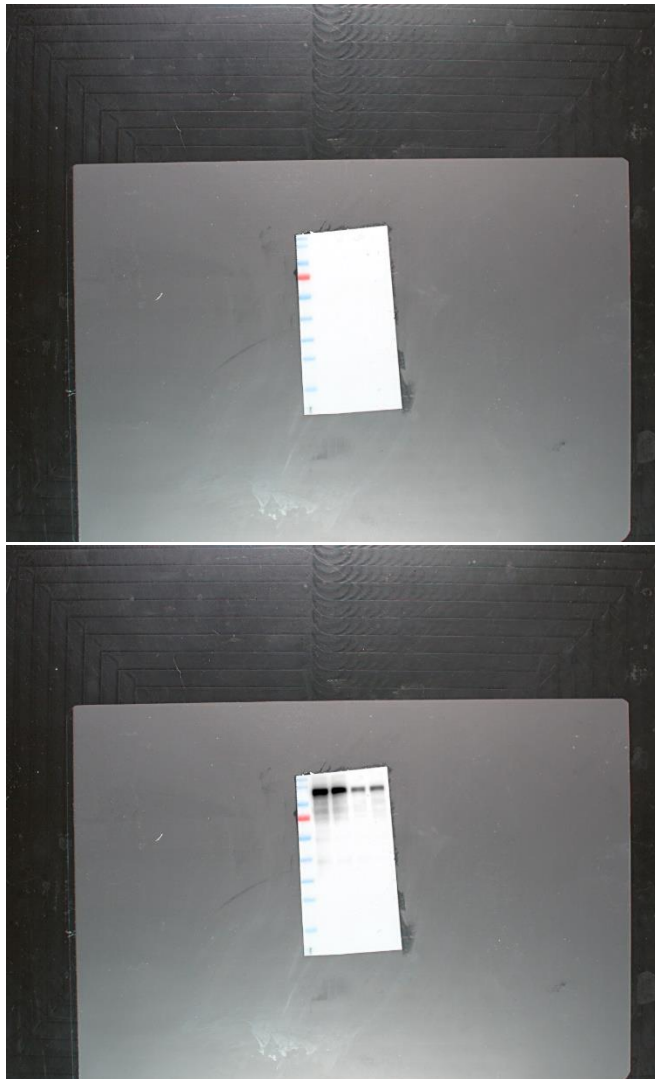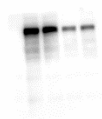

N-Ca

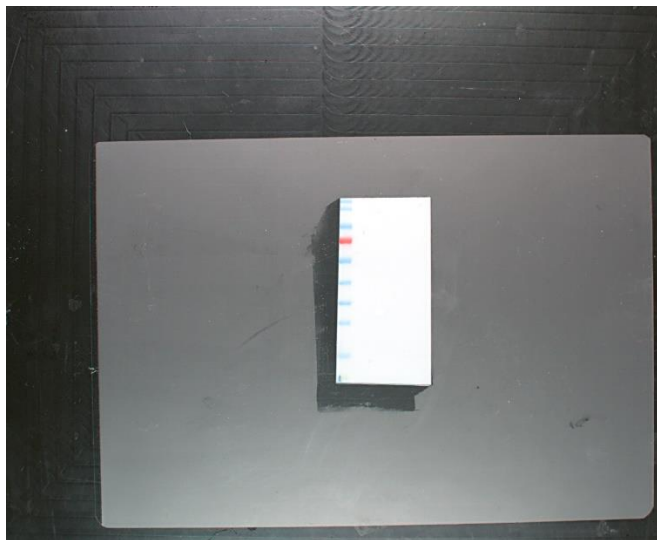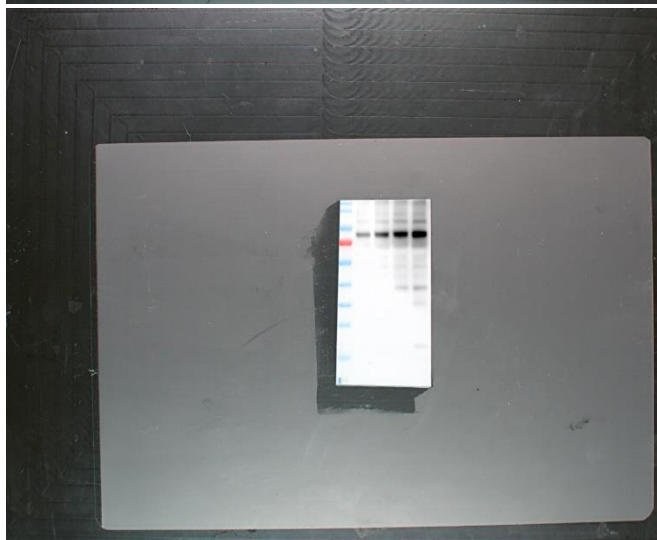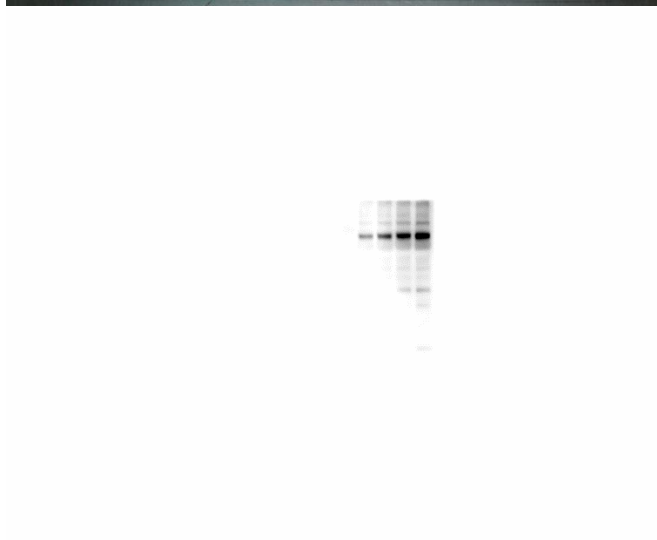

VIM

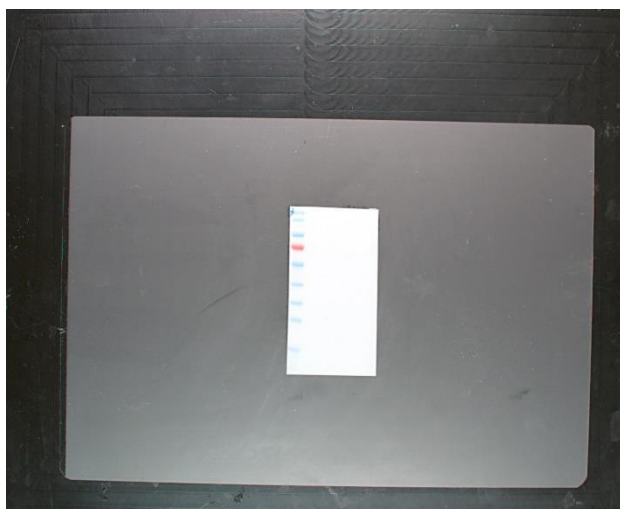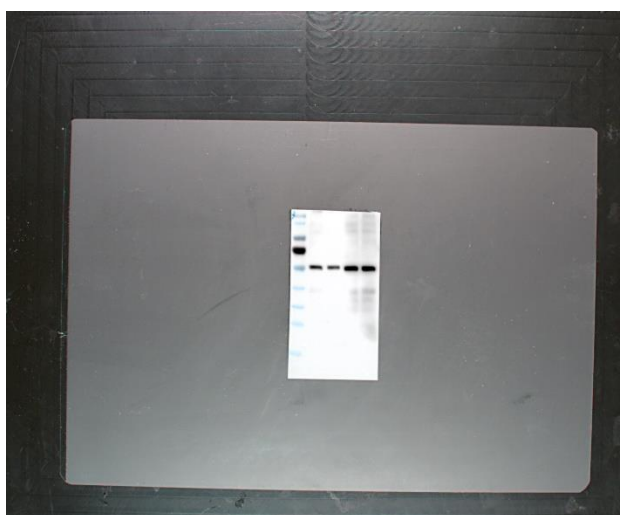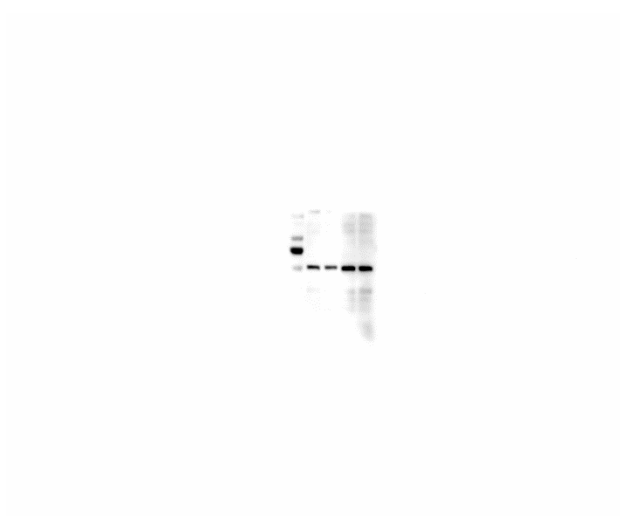

GAPDH

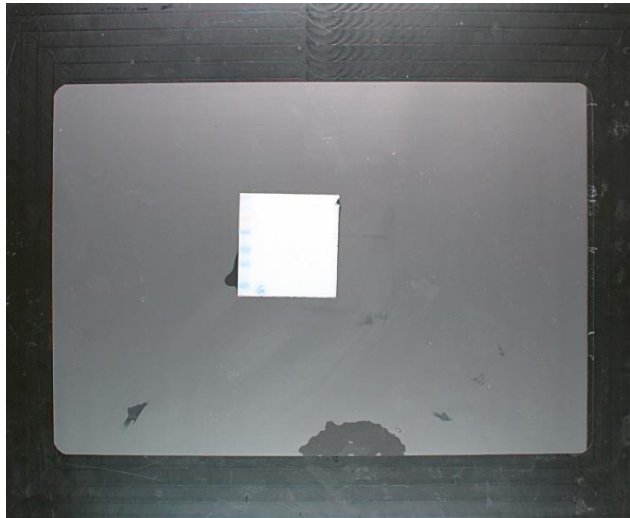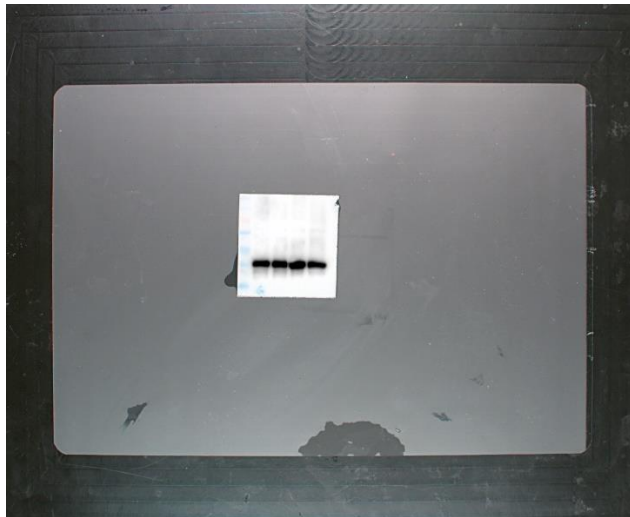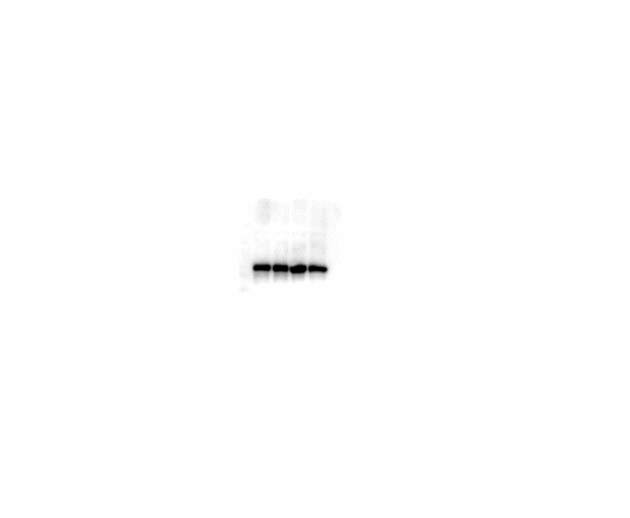

Fig 5F

PI3K

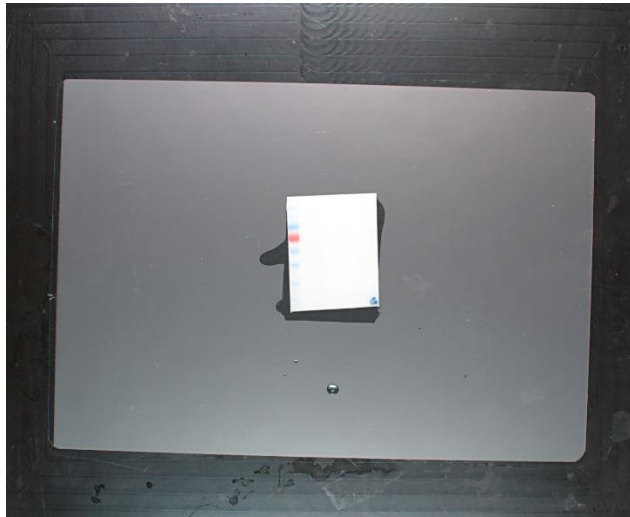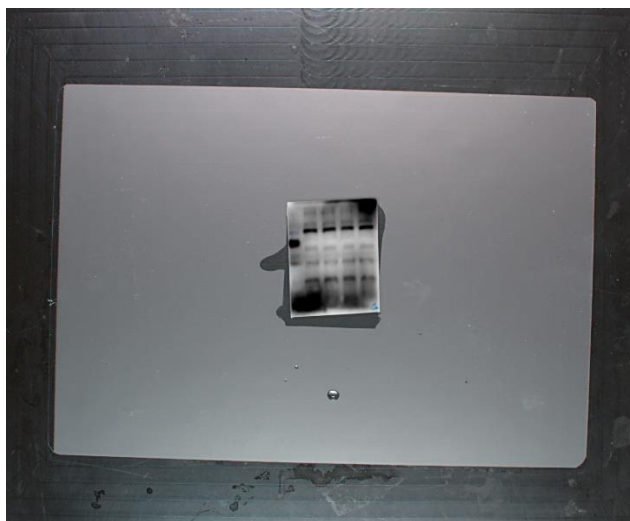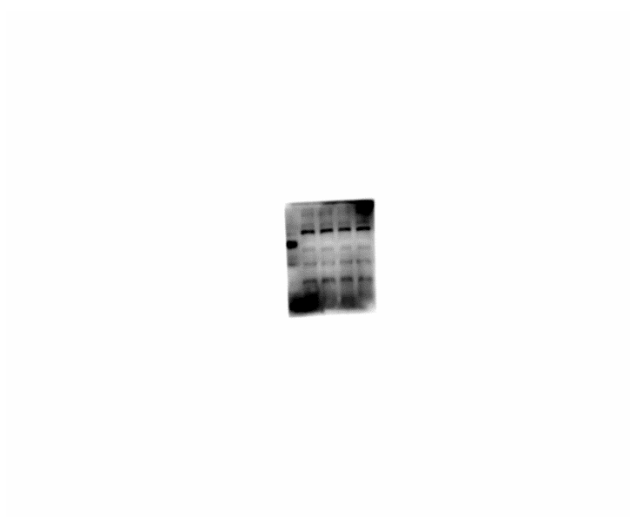

p-PI3K

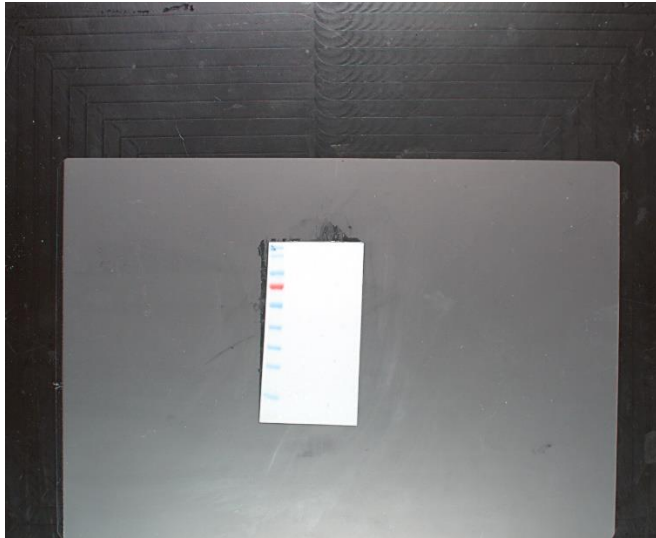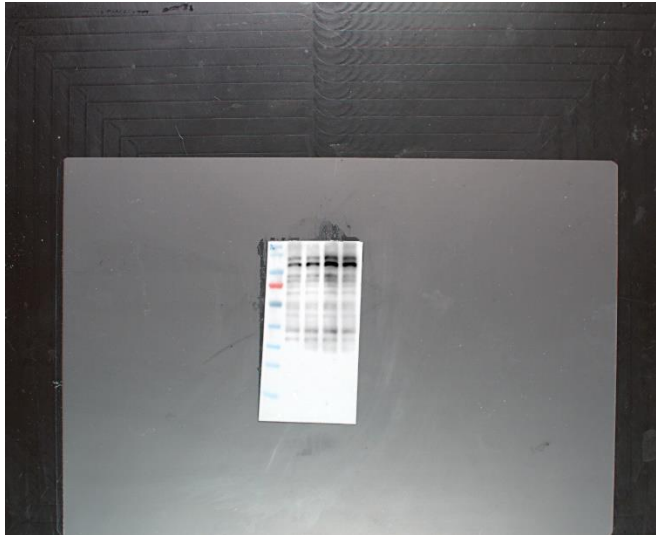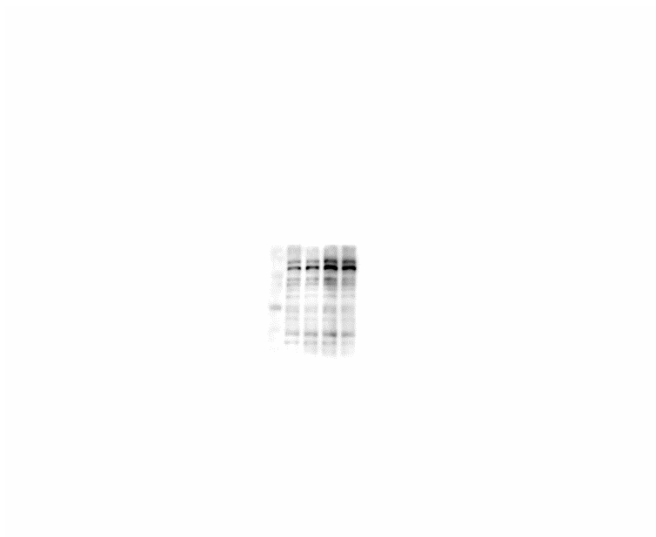

AKT

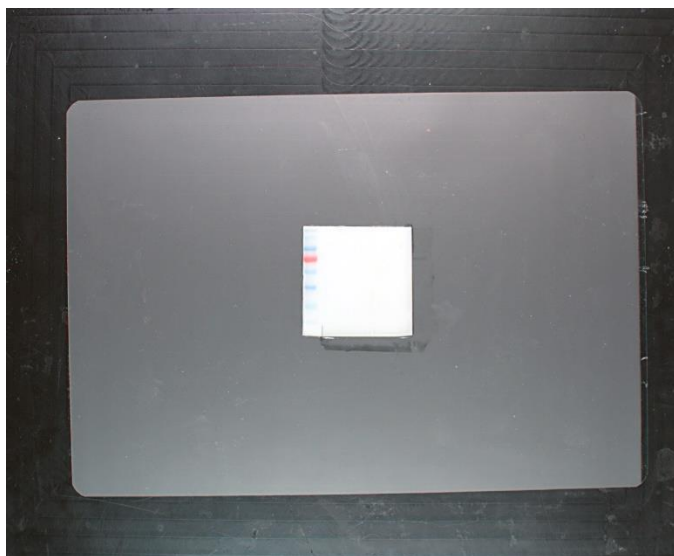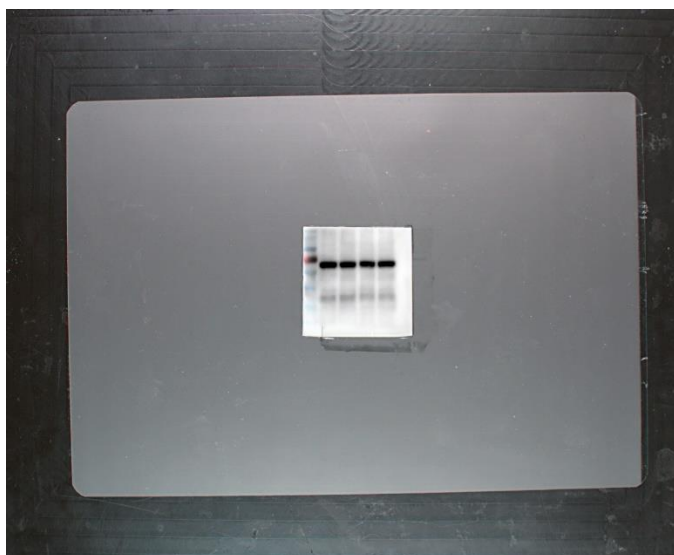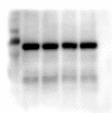

p-AKT

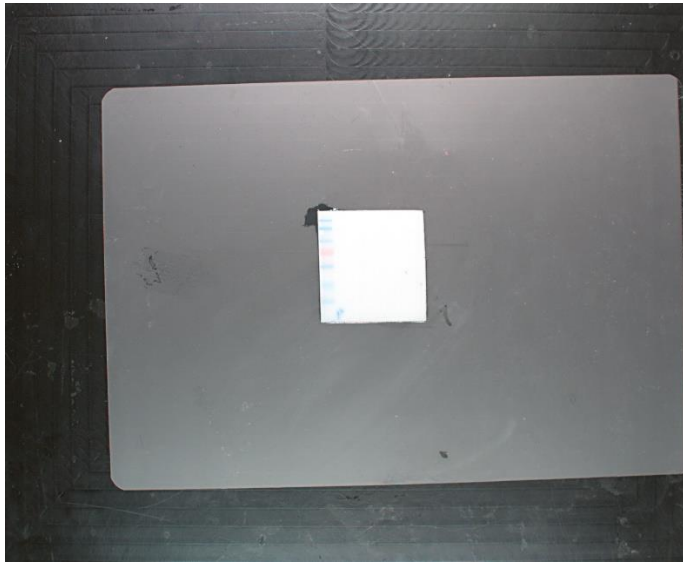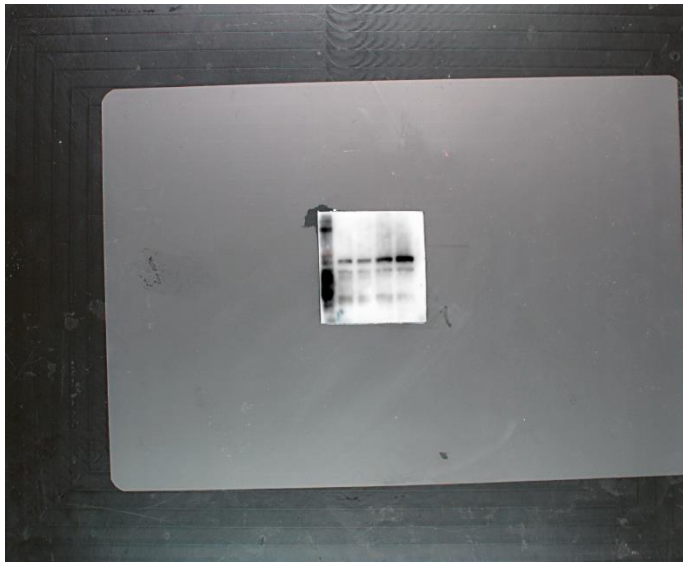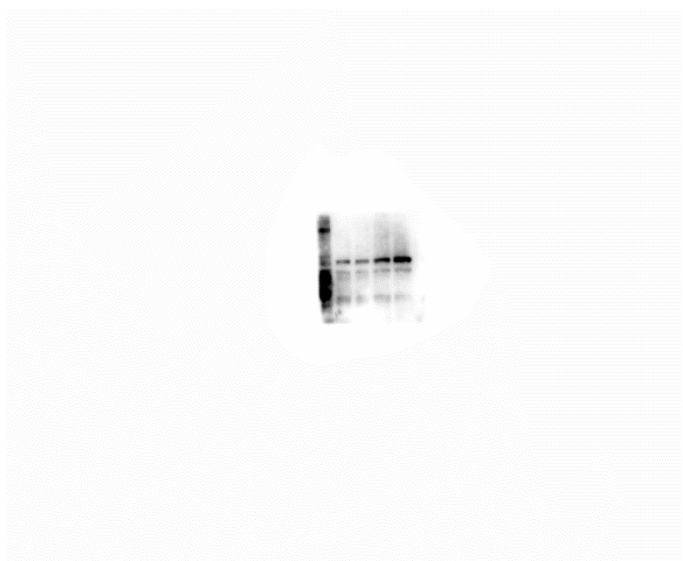

GAPDH

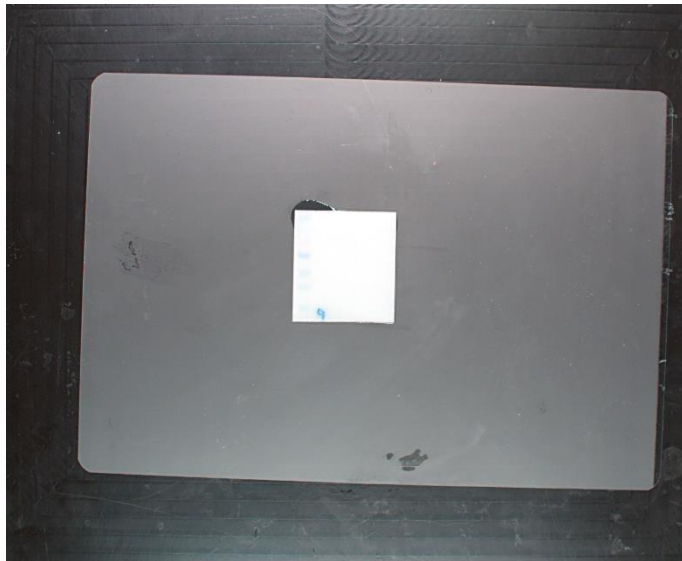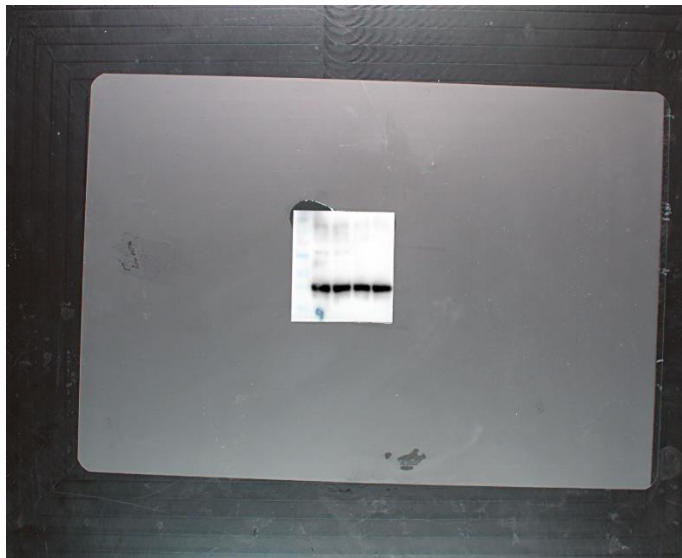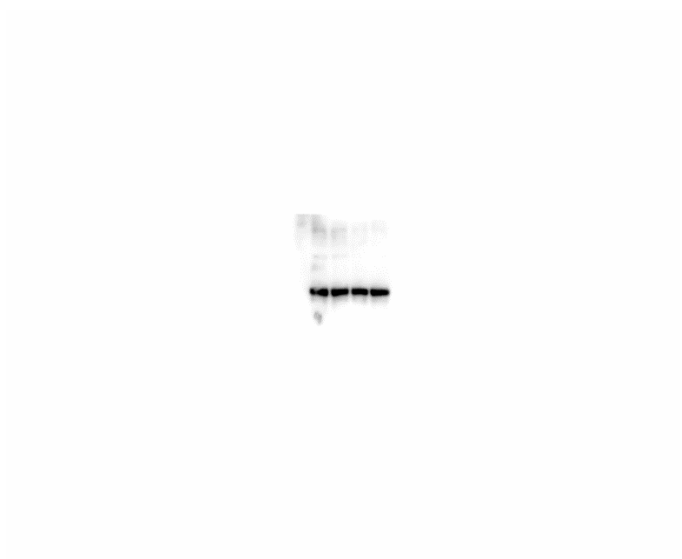

Supplement: Supplementary file 2 — Supplementary Information. [file 41598_2023_40404_MOESM2_ESM.pdf]
